# Coordination between cell proliferation and apoptosis after DNA damage in *Drosophila*

**DOI:** 10.1038/s41418-021-00898-6

**Published:** 2021-11-25

**Authors:** Mireya Ruiz-Losada, Raul González, Ana Peropadre, Alejandro Gil-Gálvez, Juan J. Tena, Antonio Baonza, Carlos Estella

**Affiliations:** 1 Centro de Biología Molecular “Severo Ochoa”, CSIC-UAM, C/Nicolás Cabrera 1, 28049 Madrid, Spain; 2 Department of Biology, Faculty of Sciences, Universidad Autónoma de Madrid, C/Darwin 2, 28049 Madrid, Spain; 3 Centro Andaluz de Biología del Desarrollo (CABD), CSIC-Universidad Pablo de Olavide-Junta de Andalucía, Seville, Spain; 4 Present Address: Centre for Organismal Studies (COS), Heidelberg University, Im Neuenheimer Feld 230, 69120 Heidelberg, Germany

**Keywords:** Cell biology, Development, Gene regulation, Molecular biology

## Abstract

Exposure to genotoxic stress promotes cell cycle arrest and DNA repair or apoptosis. These “life” or “death” cell fate decisions often rely on the activity of the tumor suppressor gene *p53*. Therefore, the precise regulation of p53 is essential to maintain tissue homeostasis and to prevent cancer development. However, how cell cycle progression has an impact on p53 cell fate decision-making is mostly unknown. In this work, we demonstrate that *Drosophila* p53 proapoptotic activity can be impacted by the G2/M kinase Cdk1. We find that cell cycle arrested or endocycle-induced cells are refractory to ionizing radiation-induced apoptosis. We show that p53 binding to the regulatory elements of the proapoptotic genes and its ability to activate their expression is compromised in experimentally arrested cells. Our results indicate that p53 genetically and physically interacts with Cdk1 and that p53 proapoptotic role is regulated by the cell cycle status of the cell. We propose a model in which cell cycle progression and p53 proapoptotic activity are molecularly connected to coordinate the appropriate response after DNA damage.

## Introduction

The ability of a cell to sense and to respond to DNA damage is essential to maintain its genetic material and tissue homeostasis. The DNA damage response (DDR) pathway has evolved in eukaryotes to preserve genomic integrity through a set of cellular responses that include cell cycle control, DNA repair, and apoptosis [1]. Cell cycle regulation is an important response, as it allows the DNA repair mechanisms to prevent the incorrect transmission of genetic material, and therefore cancer susceptibility [2]. Alternatively, if too much damage has been sustained, activation of cell death processes must occur to get rid of defective cells [3]. Although the molecular mechanisms that control these cellular responses after DNA damage have been extensively studied separately, much less is known about how these processes are coordinated to maintain tissue homeostasis. Moreover, how the progression of the cell cycle has an impact in the ability of cells to activate the apoptotic response is mostly unexplored.

DNA lesions, such as double strand breaks (DSBs), are recognized by the MRE11–RAD50–NBS1 (MRN) protein complex that recruits and activates the ataxia-telangiectasia mutated (ATM) and ATM- and Rad3-related (ATR) kinases [4]. Activated ATM and ATR phosphorylate a number of substrates, such as the histone H2AX (H2Av in *Drosophila*) and the downstream kinases Chk1 and Chk2, which are responsible for the cell cycle checkpoint and apoptotic induction [5–7].

Central in the DDR pathway is the tumor suppressor transcriptional protein p53, which can promote cell cycle arrest, DNA damage repair, apoptosis, and senescence [8]. Mammalian p53 is activated by ATM and Chk2, and in turn p53 activates the expression of numerous target genes including the cell cycle regulator p21, the DNA repair protein Rad51, or the proapoptotic genes *puma* and *noxa* [9–13]. The cellular context, timing, and extent of the activation of the DDR pathway are responsible for the fate of the cell [14]. In this sense, p53 activation and function requires a complex repertory of posttranslational modifications and protein interactions [15, 16]. However, how p53 orchestrates these cell survival and cell death responses is largely unknown.

*Drosophila* has been widely used to study the DDR, as orthologs for many of the pathway components have conserved roles [17, 18]. DSBs generated by ionizing radiation (IR) activate the ATM/ATR kinases and induce the phosphorylation of the H2Av, the cell cycle arrest through the ATR/Mei-41 and Chk1/Grapes axis, and the apoptotic response mediated by the ATM/Tefu and Chk2/Mnk branch [19–22]. As in mammals, *Drosophila* p53 is activated by Chk2 and triggers the activation of the proapoptotic genes *reaper* (*rpr*), *head involution defect* (*hid*), and *grim*. However, in *Drosophila*, p53 is dispensable for IR-induced cell cycle checkpoint [19, 23–26]. While p53 is required for the rapid IR-induced apoptosis, a p53-independent cell death that depends on c-Jun N-terminal kinase pathway activation and E2f1 helps maintain genome integrity [27–30].

In mammals and *Drosophila*, IR-induced apoptosis depends on the cell context and proliferation status of the cell [31–37]. Most IR-resistant tissues are differentiated and non-proliferative cells. Therefore, identifying the molecular determinants that regulate apoptotic induction and its connection with the cell cycle machinery is essential to understand how cells coordinate the different responses after DNA damage [38–40].

Here, we use the *Drosophila* wing imaginal disc as a model to study how cell cycle progression impact in the ability of cells to undergo DNA damage-induced apoptosis. We demonstrate that cell cycle arrested and endocycle-induced cells are insensitive to IR-induced apoptosis. We found that p53 proapoptotic activity is compromised in cell cycle arrested cells. Consistent with this, we show that p53 ability to bind to the regulatory regions of the *rpr* and *hid* genes is reduced in experimentally arrested cells. Moreover, we found that p53 and the G2/M promoting factor Cdk1 physically interact and that modification of Cdk1 activity influences p53 regulation of IR-induced apoptosis. We propose a model in which cell cycle progression and p53 activity are molecularly connected to coordinate the proapoptotic induction after DNA damage.

## Results

### Temporal dynamics of cell proliferation and apoptosis after DNA damage

**Fig. 1:**
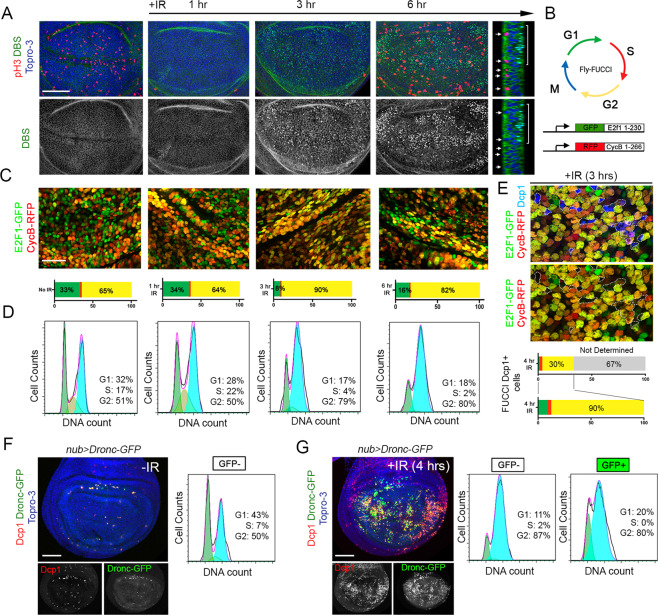
Temporal dynamics of cell cycle progression and apoptosis after IR. **A** Third instar wing imaginal discs expressing the Drice-based sensor to follow apoptotic cells (DBS, in green) and stained with the mitotic marker pH3 (red) and Topro-3 (blue) to mark the nuclei. A representative example of non-irradiated and irradiated discs dissected 1, 3, and 6 h after treatment is shown. A Z section of a wing imaginal disc dissected 6 h after IR is also show. Note that mitotic cells (red and arrows) are in a different plane and do not activate the DBS sensor (bracket). Scale bar: 50 μm. **B** Representation of the different phases of the cell cycle labeled with the Fly-FUCCI reporters (UAS*-GFP-E2F11-230* and UAS*-mRFP1-NLS-CycB1-266*) and pH3 staining (blue). Cells are labeled in green (GFP+) in the G1 phase, in red (RFP+) during the S phase, in yellow (GFP+RFP+) in the G2 phase, and in blue in mitosis. **C** Third instar wing imaginal discs from the same treatment as in **A**, expressing the Fly-FUCCI transgenes under the *ap-Gal4* driver. The quantification of the number of cells that are GFP+, RFP+, and yellow (GFP+RFP+) for each condition is indicated below each image. Scale bar: 10 μm. **D** Bottom panels show cell cycle analysis by quantification of DNA content of control and irradiated wing imaginal discs dissected 1, 3, and 6 h after treatment. **E** Wing imaginal disc cells dissected from a 3 h irradiated larvae expressing the Fly-FUCCI transgenes and stained with Dcp1 (blue). Dying cells are marked in blue and surrounded by a white dotted line. The quantification of the number of cells that are GFP+, RFP+, and yellow (GFP+RFP+) is indicated below. Apoptotic cells with very low or undetectable Fly-FUCCI reporters are considered as not determined and not quantified. Expression of an IR-induced Dronc-GFP variant with the *nubbin* (*nub*)-*Gal4* line in control (**F**) and 4 h irradiated discs (**G**). Dcp1 (red), GFP (green), and Topro-3 (blue). Note that the IR activation of Dronc leads to the appearance of GFP-positive cells in Dcp1 positive cells. Also shown is the flow cytometry analysis of DNA content in GFP-positive and GFP-negative cells in control (**F**) and irradiated discs (**G**) of the indicated genotypes. Only GFP-negative flow cytometry events were plotted in **F** as very few GFP-positive cells are present in non-irradiated discs. Scale bar: 50 μm.

To study the dynamics of cell cycle progression and apoptosis after IR, we used the highly proliferative mono-layered epithelium of the *Drosophila* wing imaginal disc (Fig. 1A). Exposure of wing imaginal cells to IR induces a rapid cell cycle arrest and the activation of the apoptotic program [19, 22, 41, 42]. To monitor cell cycle dynamics, we used the Fly-FUCCI system, fluorescence-activated cell sorting (FACS) to measure DNA content and phospho-histone H3 (pH3) staining to visualize mitosis. The Fly-FUCCI system is based on fluorochrome-tagged degrons from the cyclin B and E2F1 proteins that are degraded during mitosis and G1 or at the onset of the S phase, respectively [43] (Fig. 1B). Apoptotic cells were labeled with the effector caspase reporter DBS (for Drice-based sensor) [44]. As early as 1 h after IR, there was a strong reduction in the number of mitotic cells, although no significant changes in the fraction of cells in G1 and G2 were observed (Fig. 1A–D). Three hours after IR, cells accumulated in G2 and a dramatic increase of apoptotic cells was detected. At 6 h after IR, the G2/M mitotic arrest was already lifted as visualized by the recovery of pH3-positive cells and a high number of apoptotic cells were labeled (Fig. 1A–D). Interestingly, most of the apoptotic cells were pH3 negative.

We used the Fly-FUCCI system to visualize the phase of the cell cycle where irradiated cells die. Apoptotic cells, identified by the effector caspase Dcp1, show very low or undetectable levels of the FUCCI reporters making it difficult to determine their exact cell cycle phase. However, in those cases in which we were able to faithfully detect the reporters, most of Dcp1 positive cells (90%) were in G2 (Fig. 1E). FACS of apoptotic cells using an IR GFP inducible version of the initiator caspase Dronc [45] confirmed this result. Importantly, ~80% of Dronc-GFP-positive cells were accumulated in G2 at 4 h after IR (Fig. 1F, G).

These observations indicate that after IR, a fast but transient G2/M arrest is rapidly activated followed by apoptosis induction.

### Cell cycle arrest blocks DNA damage-induced apoptosis in wing imaginal discs

**Fig. 2:**
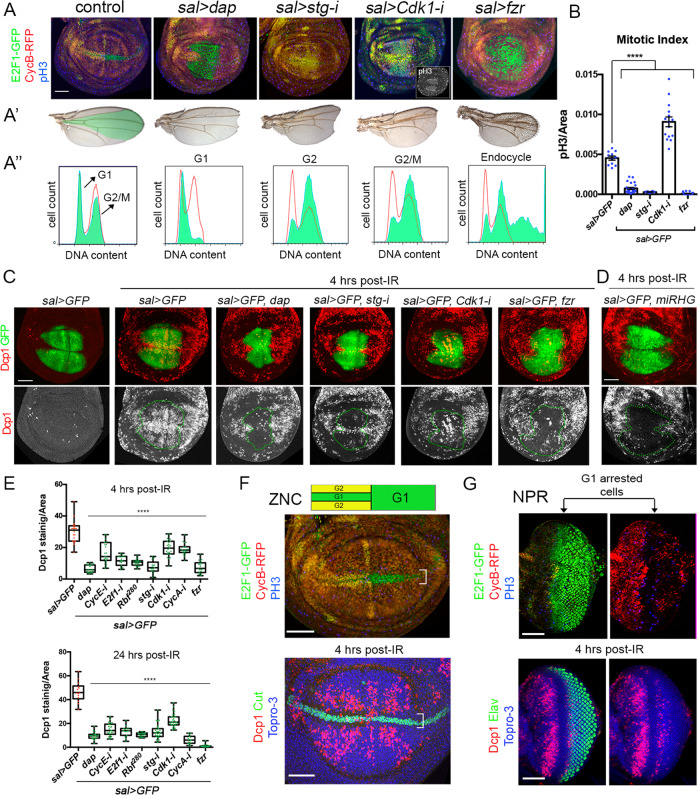
Cell cycle arrested and endocycle-induced cells attenuate IR-induced apoptosis. **A** Cell cycle perturbations by the expression of *dap*, *stg-RNAi (stg-i)*, *Cdk1-RNAi* (*Cdk1-i*), and *fzr* with the *sal-Gal4* (*sal*>) driver. The Fly-FUCCI system (*ubi-GFP-E2F11-230* and *ubi-mRFP1-NLS-CycB1-266*) and pH3 staining (blue) were used to visualize the cell cycle. The inset of the *sal*>*Cdk1-i* panel also shows the separate channel for the pH3 staining (white). The adult wing phenotypes of these experiments are shown in A’ where the *sal* domain is colored in green in the control. Cell cycle profiles of dissociated wing imaginal discs expressing the indicated cell cycle regulators and GFP in the *sal* domain is shown in A”. Red profiles correspond to control GFP-negative cells and green profiles belong to GFP-positive cells in control and cell cycle perturbed cells. **B** Mitotic index measured as the number of pH3-positive cells per area in the *sal* domain of control (*sal*>*GFP*) and in cell cycle arrested cells and endocycle-induced cells. *n* > 11 discs per genotype. Error bars indicate standard error of the mean (SEM). ****P value < 0.0001 by one-way ANOVA when compared the mean of each column with the mean of the control. **C**, **D** GFP (green) and Dcp1 staining (red and white) in wing imaginal discs expressing the indicated transgenes by the *sal-Gal4* driver in control discs and irradiated discs analyzed 4 h later. Below each panel, the Dcp1 channel is shown and the *sal* domain is outlined by green dotted lines. **E** Quantification of Dcp1 staining in the *sal* domain in wing imaginal discs expressing the indicated transgenes by the *sal-Gal4* in irradiated discs analyzed 4 and 24 h after treatment. Error bars indicate the minimum and maximum point for each genotype. Individual wing discs measurements are shown. *n* > 15 discs per genotype. ****P value < 0.0001 by one-way ANOVA when compared the mean of each column with the mean of the control (*sal*>*GFP*). **F** Scheme of the zone of nonproliferating cells (ZNC) of the wing imaginal disc and a wing carrying the Fly-FUCCI transgenes and stained with pH3 (blue) to label cells in G1 (green), G2 (yellow), S (red), and M (blue) phases. Below, a third instar wing imaginal disc from irradiated larvae dissected 4 h later. Dcp1 is in red, Cut in green, and Topro-3 in blue. The brackets indicate the ZNC were cells are arrested in G1 and G2. **G** Nonproliferating region (NPR) of the eye-antenna imaginal disc stained with Fly-FUCCI and pH3. Cells in G1 (green), S (red), G2 (yellow), and M (blue) phases are labeled. Below, an eye-antenna imaginal disc from irradiated larvae dissected 4 h later. Dcp1 is in red, Elav in green, and Topro-3 in blue. Note the absence of Dcp1 staining in the NPR (arrows) where cells are arrested in G1. Scale bar: 50 μm. See also Figs. S1 and S2.

To explore the relationship between cell cycle progression and cell death, we used the UAS/Gal4 system to arrest cells in the *spalt* (*sal*) domain of the wing pouch at different phases of the cell cycle and study their effects on IR-induced apoptosis. To precisely stage the phase of the cycle in which the cells are accumulated, we used the Fly-FUCCI system, pH3 staining, and FACS. Based on these markers, the overexpression of the *p21* ortholog, *dacapo* (*dap*), the downregulation of CycE or E2F1, and the ectopic expression of an activated form of *Retinoblastoma* (*Rbf*^*280*^) arrested cells in G1 (Fig. 2A, B and Fig. S1). A G2 and G2/M stalling was achieved by the knockdown of the Cdc25 phosphatase String (Stg) and the downregulation of the M phase promoting factor Cdc2/Cdk1, respectively (Fig. 2A, B). In addition, the overexpression of fizzy-related (Fzr/Cdh1) or the downregulation of CycA induces the transition from a mitotic cycle to an endocycle (Fig. 2A, B and Fig. S1). The endocycle is a modified cell cycle that alternates G and S phases without entering mitosis through the downregulation of Cdk1 activity [46]. All these cell cycle modifications are consistent with previous reports [47–53]. Some of these cell cycle alterations produce a moderate increase of apoptotic cells in non-irradiated discs (Fig. S2). Remarkably, IR-induced apoptosis is strongly attenuated in cell cycle arrested and endocycle-induced cells in the *sal* domain at 4 and 24 h after treatment (Fig. 2C, E and Fig. S2). These results were confirmed by the TUNEL assay, which measures DNA fragmentation caused by cell death (Fig. S2). This apoptotic induction depends on the activity of the proapoptotic genes, as the expression of a UAS transgene that simultaneously inhibit the *rpr*, *hid*, and *grim* genes (UAS*-miRHG*) abolished cell death (Fig. 2D).

Next, we analyzed the apoptotic response to IR in developmentally arrested cells. We focus on the zone of nonproliferating cells that coincides with the wing margin of the disc and in a specific region of the eye disc, known as the non-proliferative region, where cells are arrested in G1 [54, 55] (Fig. 2F, G). Our results confirm previous reports that showed that IR-induced apoptosis is strongly suppressed in any of these developmentally arrested regions [31, 56] (Fig. 2F, G).

These results demonstrated that the apoptotic response after IR is compromised in both experimentally induced and developing cell cycle arrested cells.

### Analysis of the DDR pathway in cell cycle arrested and endocycle-induced cells after IR

**Fig. 3:**
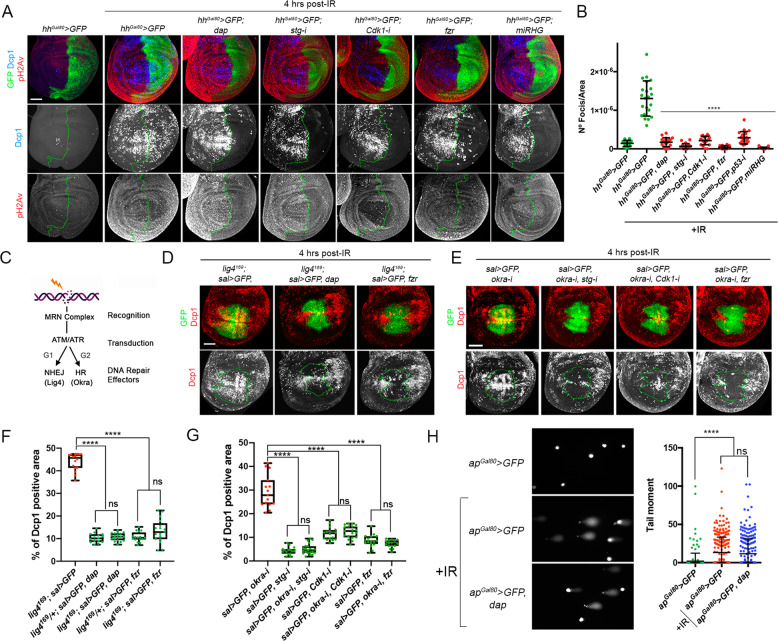
DDR pathway analysis in cell cycle arrested and endocycle-induced cells of irradiated wing imaginal discs. **A** Wing imaginal discs expressing the indicated transgenes by the *hh-Gal4, tub-Gal80*^*ts*^ (*hh*^*Gal80*^>) subjected to IR and analyzed 4 h after treatment. Imaginal discs are stained with pH2Av (red and white), Dcp1 (blue and white) and GFP (green). The antero-posterior compartment boundary is marked by a green dotted line. Wing discs were dissected from larvae raised at 31 °C for 24 h. Scale bar: 50 μm. **B** Scatter plots showing the number of pH2Av foci per area from control (non-irradiated) and irradiated imaginal discs expressing the different transgenes as in **A**. The average, standard deviation (SD) and individual measurements are shown. *n* > 14 discs per genotype. ****P value < 0.0001 by one-way ANOVA when compared the mean of each column with the mean of the control (*sal*>*GFP* IR). **C** Simplified representation of the DDR pathway and the DNA repair mechanisms activated by DSB. The MRN complex recognizes DNA lesions and their repair is mediated by ATM/ATR through an error-prone non-homologous end-joining (NHEJ) and an error-free homologous recombination (HR). NHEJ is executed by Lig4 and preferentially operates in G1, while HR takes place in S and G2 where the sister chromatid is used as a template by DNA repair proteins such as Rad54/Okra [18, 85, 86]. **D** Wing imaginal discs analyzed 4 h after IR treatment expressing the indicated transgenes by the *sal-Gal4* driver in a *lig4*^*169*^ mutant background. *dap* and *fzr* expression were selected as they arrested cells in G1 or induced the endocycle, respectively. GFP is green and Dcp1 staining is red or white. Below each panel, the Dcp1 channel is shown and the *sal* domain is outlined by green dotted lines. Scale bar: 50 μm. **E** Wing imaginal discs analyzed 4 h after IR treatment expressing the indicated transgenes by the *sal-Gal4*. *stg-i* and *Cdk1-i* arrested cells in G2 and G2/M, respectively, while *fzr* induced the endocycle. GFP is green and Dcp1 staining is red or white. Below each panel, the Dcp1 channel is shown and the *sal* domain is outlined by green dotted lines. Scale bar: 50 μm. **F**, **G** Quantification of Dcp1 staining in the *sal* domain of wing imaginal discs of the corresponding genotypes shown in **D** and **E**. Error bars indicate the minimum and maximum point for each genotype. Individual wing discs measurements are shown. *n* > 15 discs per genotype. ****P value < 0.0001 by one-way ANOVA when compared the mean of each experiment with the mean of the corresponding control. ns not significant. Note that cell cycle arrested and endocycle-induced cells attenuate apoptosis in *lig4*^*169*^ mutants and Okra depleted cells to the same extent as their controls, *lig4*^*169*^*/+*, and animals without the *okra-i* line, respectively. **H** Representative images of individual wing disc cells expressing for 24 h *GFP* or *GFP* and *dap* under the *ap-Gal4, Gal80*^*ts*^ driver (*ap*^*Gal80*^>) in control and irradiated discs. Imaginal discs were subjected to the Comet assay 5 h later. The tail DNA moment quantification for wing cells of the indicated genotypes is shown. Each dot represents a single cell. >250 comets were analyzed for each genotype and condition. Statistically significant differences based on Student’s *t* test are indicated: ****P < 0.0001 and not significant (ns). See also Fig. S3.

To analyze whether the attenuation of IR-induced apoptosis in cell cycle arrested cells is caused by a defect in the activation of the DDR pathway, we study ATM/ATR activity through pH2Av staining. We used the *hedgehog (hh)* driver in combination with the *tub-Gal80*^*ts*^ (*hh*^*Gal80*^>) to spatially and temporally express *dap, stg-i*, *Cdk1-i*, and *fzr* in the posterior compartment. In control discs 4 h after IR, an elevated number of strong nuclear pH2Av foci are readily detected accompanied with an increase in the overall staining of wing cells (Fig. 3A). These strong pH2Av foci are always associated with apoptotic cells in control and irradiated discs (Fig. S3) [57]. Consequently, the number of pH2Av foci in irradiated discs was reduced when the apoptotic pathway is compromised by the expression of the UAS*-miRHG* or by the knockdown of p53 as previously reported [57] (Fig. 3A, B). In irradiated cell cycle arrested or endocycle-induced cells, the number of pH2Av foci was also dramatically decreased (Fig. 3A, B). Notably, the background levels of pH2Av staining were also slightly reduced in irradiated cell cycle arrested cells, especially for the *dap* or *stg-i* expressing discs, when compared to the corresponding anterior control cells (Fig. 3A).

The reduction of pH2Av staining after IR could suggest that DNA lesions are being repaired in cells that have been permanently arrested or shifted toward an endocycle, and thus explains the attenuation of the apoptotic pathway. To test this possibility, we also knocked down specific components of the DDR pathway and DNA repair machinery (Fig. 3C). Importantly, the ability to attenuate apoptosis after IR in these cell cycle altered cells is maintained even when the recognition of DSB or the DNA repair mechanisms were compromised (Fig. 3D–G and Fig. S3). Similar results were obtained in a *mei-41/ATR* mutant background or in a knockdown of Tefu/ATM activity (Fig. S3).

In addition, we measured IR-induced DNA lesions using the comet assay in control and cell cycle arrested cells. Comet’s DNA tail provides information about the extent of DNA lesions and is represented as the tail moment. After IR, DNA damage is observed at the same extent in control proliferating and cell cycle arrested imaginal discs (Fig. 3H).

In summary, these results strongly suggest that the inhibition of IR-induced apoptosis observed in cell cycle arrested and endocycle-induced cells is not due to the prolonged repair of DNA lesions.

### The proapoptotic activity of p53 is compromised in cell cycle arrested cells and endocycle-induced cells

**Fig. 4:**
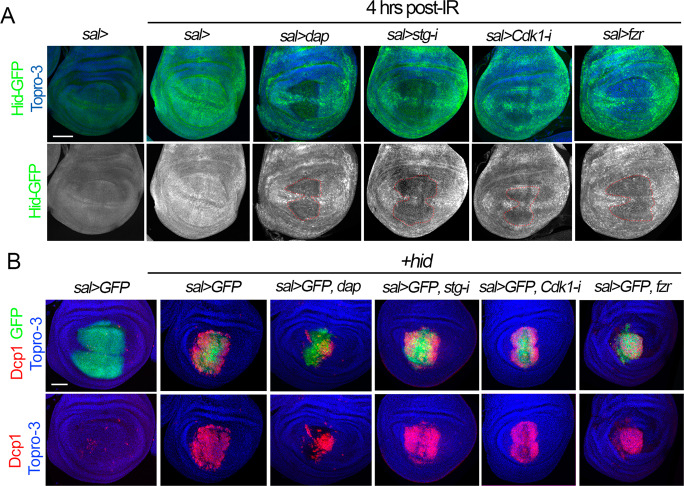
Analysis of Hid and Dcp1 activation in cell cycle arrested and endocycle-induced cells. **A** Third instar wing imaginal discs of the indicated genotypes that also express a GFP-tagged form of Hid protein (green and white). Note that after 4 h of IR, the levels of Hid-GFP increased significantly compared to a non-irradiated control. However, in cell cycle arrested and endocycle-induced cells in the *sal* domain, Hid levels are downregulated. Hid separate channel is shown below (white) and the *sal* domain is marked by red dotted lines. Topro-3 (blue) marks the nuclei. **B** Wing imaginal discs that express in the *sal* domain (green) the proapoptotic gene *hid* and the corresponding cell cycle alterations indicated in the genotypes above each panel. Dcp1 in red, GFP in green, and Topro-3 in blue. Note that although the ability of Hid to induce cell death is overall not altered in cell cycle arrested cells, we observed a reduction in Dcp1 activation in cells expressing *dap* when compared to the control. Scale bar is 50 μm. See also Fig. S4.

In order to understand at what level of the apoptotic pathway the cell cycle regulates IR-induced cell death, we first follow the activation of the proapoptotic gene *hid* in irradiated wing imaginal discs. Four hours after IR, the levels of a Hid-GFP-tagged protein form are clearly increased compared to non-irradiated discs (Fig. 4A). However, Hid-GFP levels are strongly downregulated in cell cycle arrested or endocycle-induced cells of irradiated wing imaginal discs (Fig. 4A). We confirmed the transcriptional repression of the *hid* gene in these experimental conditions using a specific *hid cis*-regulatory module (CRM) that is strongly induced after IR [58, 59] (Fig. S4). These results suggest that the inhibition of apoptosis is upstream of the proapoptotic genes. To confirm this hypothesis, we ectopically expressed *hid* or *rpr* in the *sal* domain in control and cell cycle arrested cells. In control discs, the expression of any of these proapoptotic genes strongly induced the activation of Dcp1. Accordingly, the cell cycle arrest or the induction of the endocycle overall did not have a strong impact on the ability of *rpr* or *hid* to induce apoptosis, confirming our previous results that demonstrated that the blockage of apoptosis must be upstream of the proapoptotic genes (Fig. 4B and Fig. S4).

**Fig. 5:**
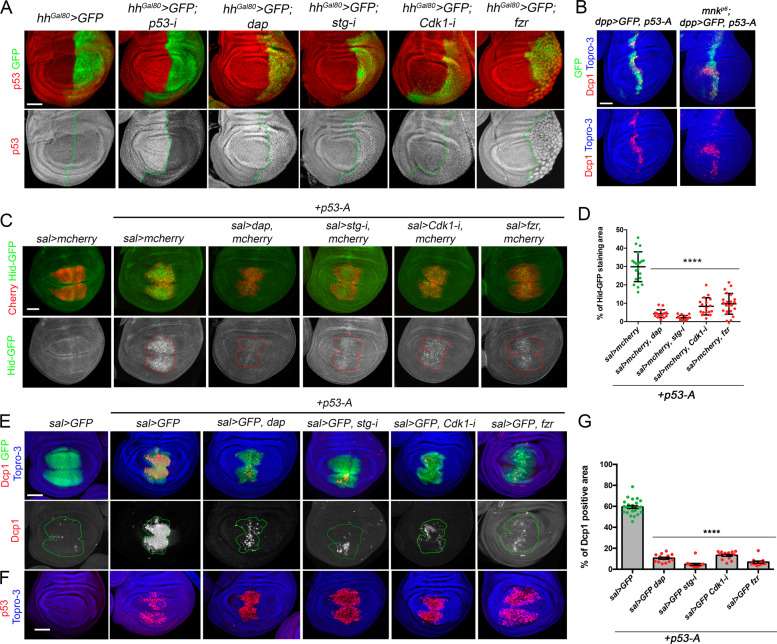
Analysis of p53 protein levels and activity in cell cycle arrested and endocycle-induced cells. **A** Third instar wing imaginal discs expressing the indicated transgene in the posterior compartment by the *hh-Gal4, tub-Gal80*^*ts*^ (*hh*^*Gal80*^>) driver and stained for p53 (red and white) and GFP (green). Separate channel for p53 is shown below each image. Note that the expression of the *p53-i* eliminates p53 staining. The gain in the p53 channel has been increased for visualization purposes. The antero-posterior compartment boundary is marked by a green dotted line. Wing discs were dissected from larvae raised at 31 °C for 24 h except for the *p53-i* that were raised all development. **B** Wing imaginal discs expressing *p53-A* under the *dpp-Gal4,* UAS*-GFP* (*dpp*>*GFP*) driver in a wild-type and *mnk*^*p6*^ mutant background stained for Dcp1 (red), GFP (green), and Topro-3 (blue). **C** Wing imaginal discs expressing the GFP-tagged form of Hid (green and white) and the indicated transgenes under the *sal-Gal4,* UAS*-mcherry* (red) driver. Separate channel for Hid-GFP is shown below each image. A dotted red line marks the *sal* domain. All the images were taken keeping the same confocal settings. **D** Scatter plots showing the quantification of Hid-GFP staining in the *sal* domain from disc expressing *p53-A* in control (*sal*>*mcherry*) and cell cycle arrested cells as indicated in **C**. The average, standard deviation (SD), and individual measurements are shown. *n* > 15 discs per genotype. ****P value < 0.0001 by one-way ANOVA when compared the mean of each column with the mean of the control (*sal*>*mcherry, p53-A*). **E**, **F** Wing imaginal discs expressing the corresponding transgenes under the *sal*>*GFP* driver. Imaginal discs were stained for Dcp1 (red and white), GFP (green), Topro-3 (blue) in **E** and for p53 (red) and Topro-3 (blue) in **F**. Separate channel for Dcp1 is shown below each image. A dotted red line marks the *sal* domain. **G** Quantification of Dcp1 staining in the *sal* domain of wing imaginal discs from the genotypes presented in **E**. Error bars indicate SEM. *n* > 11 disc per genotype. ****P value < 0.0001 by one-way ANOVA when compared the mean of each column with the mean of the control (*sal*>*GFP, p53-A*). Scale bar is 50 μm. See also Fig. S5.

Initial IR-induced cell death requires p53 activity, which activates the expression of the proapoptotic genes *rpr* and *hid* [25, 59–61]. Therefore, we decided to study the connection between cell cycle progression and p53 regulation of IR-induced apoptosis. *Drosophila* has a single *p53* family member that encodes for four isoforms. p53-A, also known as ΔNp53, is the most abundant isoform in imaginal discs and the one responsible for the IR-induced apoptosis [39, 62, 63]. First, we analyzed p53 protein levels in cells of the wing imaginal disc that have been arrested or shifted to an endocycle. No significative changes in p53 protein levels or localization were observed in experimentally arrested cells both in non-irradiated and irradiated wing discs (Fig. 5A and Fig. S5). Next, we tested whether the block on cell proliferation could have an impact on the ability of p53 to activate the expression of *hid* and to induce apoptosis. Forced expression of *p53-A* in the wing imaginal disc strongly activated *hid* expression and induced apoptosis (Fig. 5C, E). However, the overexpression of *p53-A* in cells that are simultaneously arrested or shifted toward an endocycle has a dramatically reduced ability to activate the apoptotic program (Fig. 5C–G). In these experiments, p53-A protein levels are comparable between control cycling cells and arrested cells (Fig. 5F).

The decrease in apoptotic activity of p53-A in cell cycle arrested and endocycling-induced cells is not due to defects on p53 activation by the ATM/Tefu and Chk2/Mnk axis as the overexpression of *p53-A* in an *mnk/Chk2* mutant background is able to induce apoptosis to the same extent as in a wild-type background (Fig. 5B).

Altogether, these results demonstrate that cell cycle progression regulates p53 proapoptotic activity.

### p53 proapoptotic response is controlled by the G2/M promoting factor Cdk1

**Fig. 6:**
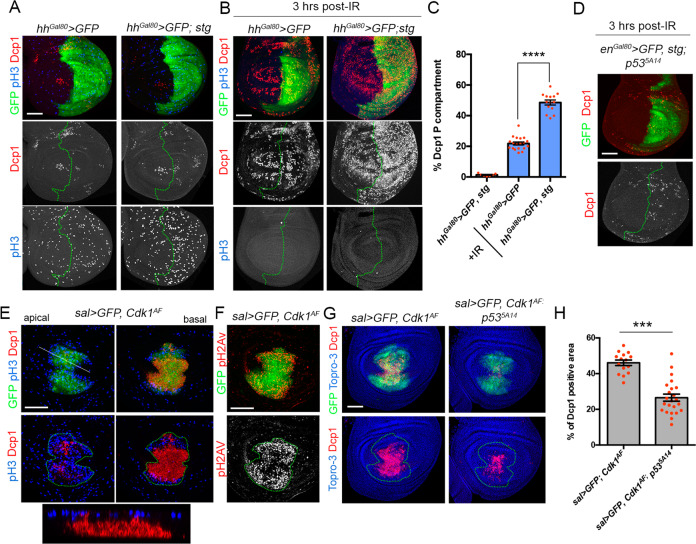
The G2/M promoting factor Cdk1 regulates p53-apoptotic response after IR. **A** Third instar wing imaginal discs expressing the corresponding transgene for 24 h under the *hh-Gal4, tub-Gal80*^*ts*^ (*hh*^*Gal80*^>) and stained for Dcp1 (red), pH3 (blue), and GFP (green). Separate channels for Dcp1 and pH3 are shown. The antero-posterior compartment boundary is marked by a green dotted line. **B** Third instar wing imaginal discs expressing the indicated transgenes for 24 h as in **A**, subjected to IR and dissected 3 h after treatment. Discs were stained for Dcp1 (red), pH3 (blue), and GFP (green). Separate channels for Dcp1 and pH3 are shown. The antero-posterior compartment boundary is marked by a green dotted line. **C** Quantification of Dcp1 staining in the *hh* domain (posterior compartment) of wing imaginal discs from the genotypes and treatments described in **A** and **B**. Error bars indicate SEM. *n* > 14 disc per genotype except for *hh*^*Gal80*^>*GFP, stg* where *n* = 7. Statistically significant differences based on Student’s *t* test are indicated: ****P < 0.0001. **D** Wing imaginal discs expressing *stg* under the *en-Gal4, tub-Gal80*^*ts*^ (*en*^*Gal80*^>) for 24 h in a *p53*^*5A14*^ mutant background. Third instar larvae were subjected to IR and dissected 3 h later. Wing discs were stained for Dcp1 (red and white) and GFP (green). Separate channels for Dcp1 are shown. The antero-posterior compartment boundary is marked by a green dotted line. Expression of the noninhibitable version of Cdk1 (*Cdk1*^*AF*^) in the *sal* domain of third instar wing discs stained for pH3 (blue), Dcp1 (red), and GFP (green) in **E** and for pH2Av (red and white) in **F**. An apical, basal, and Z section are shown in **E**. The *sal* domain is marked by a green dotted line in **E** and **F**. Separate channel for pH2AV staining is shown in **F**. **G** Third instar wing imaginal discs expressing the *Cdk1*^*AF*^ transgene under the *sal*>*GFP* driver in a control and a *p53*^*5A14*^ mutant background. Imaginal discs were stained for Dcp1 (red), Topro-3 (blue), and GFP (green). The *sal* domain is marked by a green dotted line. **H** Dcp1 staining quantification in the *sal* domain of wing imaginal discs from the genotypes indicated and described in **G**. Error bars indicate SEM. *n* > 15 disc per genotype. Statistically significant differences based on Student’s *t* test are indicated: ***P < 0.001. Scale bar is 50 μm.

Our data indicate that arresting cells at G1, G2, or the induction of the endocycle attenuate p53 proapoptotic response. In all these conditions the progression to mitosis is blocked, suggesting that p53-A proapoptotic activity could be regulated at the G2/M phase. Moreover, we have described that IR-induced apoptotic cells accumulated preferentially at the G2 phase. G2 to M transition requires the activation of Cdk1 by the phosphatase Stg that removes the inhibitory phosphates [64]. Therefore, we decided to test whether active Cdk1 could regulate p53-A proapoptotic function. We induced Cdk1 activity by the expression of *stg* in the posterior compartment and measured IR-induced apoptosis. In control discs without IR, the temporal expression of *stg* for 24 h in the posterior compartment had almost no effect on apoptosis (Fig. 6A). Remarkably, in wing discs from the same genotype dissected 3 h after IR, the expression of *stg* significantly increased the number of apoptotic cells when compared to an irradiated control disc (Fig. 6B, C). Importantly, the increase of apoptosis induced by *stg* overexpression in irradiated discs is dependent on p53 activity (Fig. 6D).

The expression of an active non-inhibitable version of Cdk1 (called Cdk1^AF^) force cells to enter mitosis and to bypass the G2/M checkpoint [65]. In addition, *Cdk1*^*AF*^ expression induced a strong apoptotic response, detected mostly in pH3 negative cells, that is associated with an increase of ATM/ATR activity visualized by pH2Av staining (Fig. 6E, F). This ectopic apoptotic induction is partially dependent on p53 activity, as Dcp1 staining was significantly reduced in the *p53*^*5A14*^ mutant background of *sal*>*Cdk1*^*AF*^ discs (Fig. 6G, H). These and previous results support the role of Cdk1 as a regulator of p53 proapoptotic activity in G2/M.

### Cdk1 regulates p53 binding to the proapoptotic genes

**Fig. 7:**
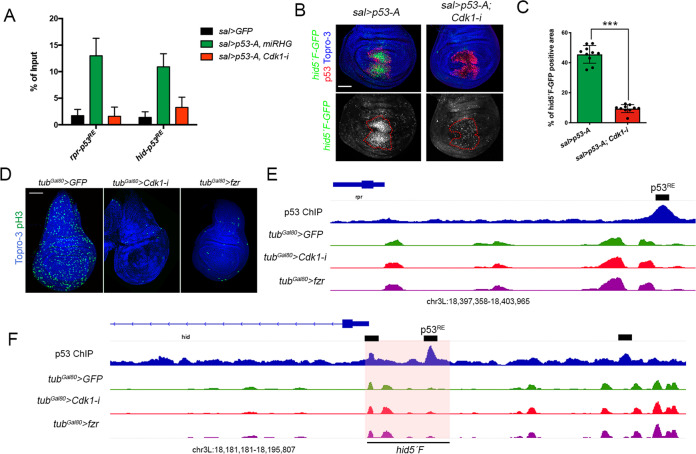
p53-A binding at the p53^RE^ of the proapoptotic genes *hid* and *rpr* and chromatin accessibility in cell cycle arrested cells. **A** Analysis of p53-A binding by chromatin immunoprecipitation experiments with anti-Myc at the p53^RE^ of the *hid* and *rpr* genes from wing imaginal discs of the following genotypes: *-sal*>*GFP*. -*sal*>*GFP*, *p53-A (Myc)*, *miRHG*. -*sal*>*GFP*, *p53-A (Myc)*, and *Cdk1-i*. Enrichment values were normalized to a “mock” sample (IgG). Error bars represent SEM of three independent experimental replicates. **B** Wing imaginal discs expressing *p53-A* or *p53-A* and *Cdk1-i* under the sal> driver. *hid 5´F-GFP* activity is in green, p53 in red, and Topro-3 in blue. Separate channel for the *hid 5´F-GFP* is presented below each image with the *sal* domain marked with a red dotted line. All the images were taken keeping the same confocal settings. **C** Quantification of *hid 5´F-GFP* staining in the *sal* domain of wing imaginal discs from the genotypes presented in **B**. Error bars indicate SEM. *n* > 10 disc per genotype. Statistically significant differences based on Student’s *t* test are indicated: ***P < 0.001. **D** Third instar imaginal discs of the genotype *tub-Gal4, tub-Gal80*^*ts*^ (*tub*^*Gal80*^>) expressing *GFP* (channel not shown), *Cdk1-i* or *fzr* for 48 h and stained for pH3 (green) and Topro-3 (blue). Note the strong reduction of mitotic cells (pH3) compared to the control (GFP). ATAC-seq genomic tracks at the *rpr* (**E**) and *hid* (**F**) locus of control proliferating wing disc cells (*tub*^*Gal80*^>*GFP*), cell cycle arrested wing cells (*tub*^*Gal80*^>*Cdk1-i*), and endocycle-induced wing cells (*tub*^*Gal80*^>*fzr*). The upper track shows the p53 genome-wide binding profile in *Drosophila* embryos as described in ref. [35]. Black bars indicate p53-binding peaks. The location of the described p53^REs^ and of the *hid 5´F-GFP* regulatory region is indicated. Scale bar is 50 μm. See also Fig. S6.

To explore how the cell cycle, and specifically Cdk1, could regulate p53 proapoptotic activity we performed a chromatin immunoprecipitation (ChIP) assay to study p53-A binding to the regulatory regions of the proapoptotic genes in Cdk1 knockdown cells. We used negative control imaginal discs (*sal*>*GFP*) and discs that overexpressed, under the *sal* driver, a Myc-tagged *p53-A* version in proliferating (*sal*>*GFP, p53-A, miRHG*) and *Cdk1-RNAi* cells (*sal*>*GFP, p53-A-Myc, Cdk1-i*) (Fig. 7A). We expressed the *miRHG* along with *p53-A* in the positive control proliferating discs to reduce the number of apoptotic cells that could interfere with the ChIP assay. Strong chromatin enrichment was observed at the p53 responding elements (p53^REs^) of the *rpr* and *hid* genes when *p53-A* was overexpressed in proliferating cells. However, this enrichment was strongly reduced in Cdk1 downregulated cells (Fig. 7A). Consistently with the ChIP assay, p53-A ability to induce the activity of two different *hid* CRMs is compromised in Cdk1 knockdown cells (Fig. 7B, C and Fig. S6) [59, 66]. Similar defects in p53-A binding and *hid* CRMs activation were observed for G1 arrested cells or endocycle-induced cells by the expression of *dap* and *fzr*, respectively (Fig. S6).

It has been proposed that endocycling cells of the salivary glands and late stage embryos are refractory to IR-induced apoptosis by an epigenetic silencing of the proapoptotic genes [39, 40]. We reason that a similar mechanism could be employed in cell cycle arrested cells and decided to explore this possibility. We used ATAC-seq to measure chromatin accessibility in experimentally cell cycle arrested and endocycle-induced cells of the wing imaginal disc. To this end, we knockdown Cdk1 levels or expressed *fzr* in all wing imaginal cells for 48 h before dissection. In these conditions, very few pH3-positive cells were observed compared to the control (Fig. 7D). Importantly, the chromatin accessibility profile at the *rpr* or *hid* locus was very similar in proliferating and Cdk1 knockdown or *fzr* expressing discs (Fig. 7E, F and Table S1). These results were confirmed when a sensor that reflects chromatin accessibility at the irradiation responsive enhancer region (IRER) locus was used [40, 67] (Fig. S6). Consistently, no changes in IRER activity were observed in cell cycle arrested cells and endocycle-induced wing cells, suggesting that chromatin accessibility at this region is not affected by the cell cycle status of the cell.

Instead, our results suggest a direct effect of Cdk1 over p53 that would regulate its binding to the regulatory regions of the proapoptotic genes

### p53 physically interacts with Cdk1 through the transactivation domain (TAD)

**Fig. 8:**
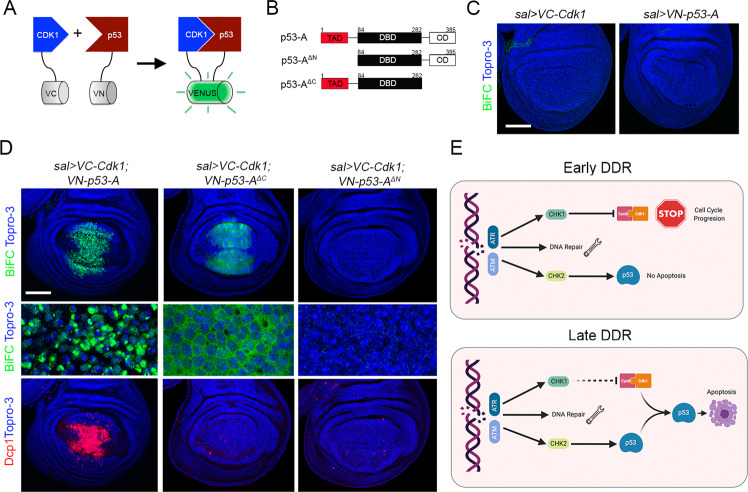
p53 physically interacts with Cdk1. **A** Cartoon illustrating the BiFC principle. No fluorescent N- and C-terminal fragments of the GFP variant Venus are fused to p53 (VN-p53) and Cdk1 (VC-Cdk1). When both proteins are co-expressed inside the cells juxtaposes the Venus fragments resulting in structural complementation and green fluorescence. This technique enables the direct visualization of protein interactions in living cells. **B** Scheme of the different p53-A protein versions generated for the BiFC analysis. Amino acid positions defining p53 domains are indicated: transactivation domain (TAD, in red), DNA-binding domain (DBD, in black), and oligomerization domain (OD, in white). **C** Third instar wing imaginal discs expressing only *VC-Cdk1* or *VN-p53-A* under the *sal-Gal4* driver did not show any BiFC signal (green). Topro-3 staining marks nuclei in blue. **D** BiFC analysis by the co-expression of *VN-p53-A, VN-p53-A*^*ΔC*^, and *VN-p53-A*^*ΔN*^ with *VC-Cdk1* under the *sal-Gal4* driver. Note that *p53-A-VN* and *VC-Cdk1* induced a strong nuclear BiFC signal (green) and the induction of apoptosis (Dcp1, red). The expression of the C-terminal deletion, *VN-p53-A*^*ΔC*^ with *VC-Cdk1* generates a cytoplasmic BiFC signal, but failed to induce cell death. In contrast, neither BiFC signal nor apoptosis was observed by the co-expression of *VC-Cdk1* with the N-terminal deletion of *p53-A* (*VN-p53-A*^*ΔN*^). A higher magnification of a region in the *sal* domain is presented. **E** Simplified representation of the DDR model. DNA damage triggers the key signal transducers ATR and ATM kinases that activate the downstream effectors Chk1 and Chk2. Chk1 stop cell cycle progression through the inactivation of Cdk1 and activates the DNA damage repair mechanisms. Chk2 induces the activation of p53, however, the proapoptotic activity of p53 is blocked in cell cycle arrested cells due to the presence of inactive Cdk1/CycB complexes. Some hours later, the G2/M blockage is progressively lifted and cells with unrepaired DNA are sent to apoptosis before entering mitosis through the activation of p53 by the ATM/Chk2 pathway and the presence of active Cdk1. Scale bar is 50 μm. See also Fig. S7.

To further understand the molecular connection between p53 and Cdk1, we tested whether these proteins physically interact and its functional relevance in regulating the apoptotic response. We used the bimolecular fluorescence complementation (BiFC) assay to evaluate the direct physical interaction between p53 and Cdk1 in wing imaginal cells [68, 69]. This method is based on the reconstitution of the Venus fluorescent protein when two non-fluorescent Venus fragments fused to the proteins of interest (in this case VC-Cdk1 and VN-p53-A) are brought together in the cell (Fig. 8A). The expression of each individual construct did not show any BiFC signal, however when VC-Cdk1 and VN-p53-A were coexpressed, a specific strong nuclear Venus signal could be detected (Fig. 8C, D and Fig. S7).

Next, we investigated which p53 domain is responsible for its interaction with Cdk1. We created p53 N- and C-terminal deletions for the TAD and the oligomerization domain, respectively (Fig. 8B). While the C-terminal deletion (VN-p53-A^ΔC^) maintains its ability to interact with VC-Cdk1 as visualized by BiFC signal, this complex is mainly observed in the cytoplasm and it is unable to trigger apoptosis (Fig. 8D). In contrast, deletion of the N-terminal domain (VN-p53-A^ΔN^) strongly abolished the p53-A/Cdk1 BiFC signal and the apoptotic induction (Fig. 8D).

Together, these results indicate that p53 can interact directly with Cdk1 through the TAD.

## Discussion

### Cdk1 connects the cell cycle with the apoptotic program

In this work, we study the connection between cell cycle progression and apoptotic induction after DNA damage. Our main conclusion is that p53 proapoptotic activity can be regulated by the G2/M promoting factor Cdk1 in response to DNA damage. In addition, we observed that late p53-independent apoptosis is also suppressed in arrested and endocycle-induced cells probably through the reduction of aneuploidy cells due to the blockage of mitosis [27, 28].

Several mechanisms have been proposed to explain the differential apoptotic sensitivity of DNA damage cells depending on their proliferating status [34, 36, 40]. This includes the epigenetic silencing at the regulatory regions of the proapoptotic genes and the proteasome-dependent degradation of p53 [39, 40]. Our results in wing disc cells demonstrate that p53 protein levels and chromatin accessibility at the *rpr* and *hid* locus are comparable in cycling and experimentally arrested or endocycle-induced cells. Instead, our experiments point to a direct regulation of p53 proapoptotic activity by Cdk1.

In human cells, p53 is specifically phosphorylated by Cdc2/Cdk1. This interaction is not only important for promoting p53 binding site preference but also to enhance p53 binding to its target sequences [70–73]. In this work, we demonstrate that p53 physically interacts with Cdk1, and that Cdk1 regulates p53 binding to the p53^RE^ of the proapoptotic genes. It would be interesting to study how Cdk1 affects the p53 transcriptional output at a molecular level. One possibility is that p53 phosphorylation by active Cdk1 enables the selection of specific targets linking cell cycle progression to p53 transcriptional output. Supporting this hypothesis, it has been shown in *Drosophila* that p53 regulates different DNA damage programs in a postmitotic tissue compared to a proliferating one through the differential p53 binding in these tissues [35].

### Life vs death decisions after DNA damage

Cells sensing DNA damage are faced with antagonizing responses such as cell cycle arrest and DNA repair or the induction of apoptosis. A central role in these prosurvival and proapoptotic cell fate decisions is played by p53 [74–77]. p53 activity is tightly regulated by posttranslational modifications, cofactor interactions, chromatin structure, and cellular context [14]. In addition, in this work we demonstrated that p53-induced apoptosis requires active Cdk1. However, we also described that wild-type cycling cells arrest in G2 and die upon IR. This G2 arrest is transient and mediated by the inhibitory Cdk1 kinase Myt1 [78]. We suggest that in these early irradiated discs, residual active Cdk1 is enough to mediate p53-dependent apoptosis but insufficient to promote G2/M transition. Once the G2 arrest is lifted, the progressive accumulation of active Cdk1 levels after IR would increase the number of apoptotic cells and trigger mitotic entry. In this sense, p53-apoptotic induction would require lower active Cdk1 levels and would be a faster response than the G2/M transition where phosphorylation of a large number and variety of proteins by Cdk1 is needed [79].

All together, we propose a model where the connection between cell cycle progression, through the regulation of Cdk1 activity, and the proapoptotic function of p53 allow cells with DNA damage to be protected from apoptotic induction when the DNA repair mechanisms are operational (Fig. 8E). After sensing DNA damage, cells activate the mitotic checkpoint, which in *Drosophila* arrest cells in G2, allowing time for the DNA repair mechanisms. This mitotic delay is dependent on ATR/Mei-41 and Chk1/Grp that transiently downregulates Cdk1 active levels [17, 21]. At the same time, in response to DNA damage ATM/Tefu phosphorylates Chk2/Mnk, which in turn activates p53 [24]. We have shown that the proapoptotic function of p53 is blocked in cell cycle arrested and endocycle-induced cells due to Cdk1 inactivation. This cell cycle arrest gives cells an opportunity to repair their DNA lesions. As the G2 arrest is progressively lifted, cells with unrepaired DNA are sent to apoptosis before entering mitosis through the activation of p53 by the ATM/Chk2 pathway and the presence of active Cdk1. The molecular connection between cell cycle progression and p53 proapoptotic activity contributes to the suppression of DNA damage-induced genomic instability.

Cell cycle arrest and apoptosis protection are hallmarks of cellular senescence [80]. Our results suggest a possible common mechanism employed by cells to prevent apoptotic induction through the regulation of the cell cycle in different stress conditions such as DNA or tissue damage and senescence [81, 82].

## Materials and methods

### *Drosophila* strains

The different *Drosophila melanogaster* lines were maintained on standard medium at 25 °C in light/dark cycles of 12 h except for the temperature shift experiments (see below). The sex of experimental larvae was only considered relevant when selecting for specific mutations that were X-linked. The strains used in this study are summarized in Table S2.

Reporters: *DBS* (*DBS-GFP*), UAS*-Dronc-GFP*, *tagged hid-GFP*, *hid*^*20-10*^*-lacZ*, *hid 5*´*F-GFP*, *IRER{ubi-DsRed}*, and the Fly-FUCCI reporters *ubi-GFP-E2F11-230* and *ubi-mRFP1-NLS-CycB1-266 (ubi-flyFUCCI)* and UAS*-GFP-E2F11-230* and UAS*-mRFP1-NLS-CycB1-266*. The Gal4 drivers: *ap-Gal4, tub-Gal80*^*ts*^ (*ap*^*Gal80*^>), *sal*^*EPv*^*-Gal4* (*sal*>), *dpp*^*blink*^*-Gal4* (*dpp*>), *hh-Gal4,* UAS*-GFP; tub-Gal80*^*ts*^ (*hh*^*Gal80*^>), *en-Gal4,* UAS*-GFP; tub-Gal80*^*ts*^ (*hh*^*Gal80*^>), *nub-Gal4* and *tub-Gal80*^*ts*^; *tub-Gal4* (*tub*^*Gal80*^>). The UAS lines: UAS*-dap*, UAS*-stg-i,* UAS*-Cdk1-i*, UAS*-fzr*, UAS*-CycE-i,* UAS*-E2f1-i,* UAS*-Rbf*^*280*^, UAS*-CycA-i,* *UAS*-mre-11-i, UAS*-okra-i,* UAS*-tefu-i,* UAS*-Dcr-2,* UAS*-rpr,* UAS*-hid,* UAS*-GFP,* UAS*-mcherry,* UAS-*p53-A-myc,* UAS*-p53;* UAS*-Su(var)3-9-i,* UAS*-miRNA-RHG,* UAS*-stg,* UAS*-CycE,* UAS*-Cdk1*^*AF*^, UAS*-p53-VN,* UAS*-Cdk1-HA,* UAS*-dac-VN* and UAS*-abdm-VC*. The following mutant lines were used: *lig4*^*169*^, *mei-41*^*D5*^, *mnk*^*p6*^, and *p53*^*5A14*^. Details for the different lines and reagents used in this study can be found in Table S2.

UAS*-Dcr2* was used in combination with different RNAi lines to enhance message knockdown.

Cdk1 downregulation shows a relative mild phenotype visualized by the stalling of cells at the mitosis phase (Fig. 2A); therefore, in Figs. 3–7, we used two different UAS*-Cdk1* lines together to increase the knockdown efficiency. Details for the different lines used in this study can be found in Table S2.

### Temperature shifts experiments

The temporal expression of the different UAS lines was restricted when needed using the Gal4/Gal80^ts^ UAS system [83]. Briefly, embryos were collected for 2 days, maintained at the restrictive temperature (17 °C) and then shifted to the permissive temperature (31 °C) for the appropriated time prior dissection.

### Imaginal discs staining, image acquisition and analysis

Third instar larvae were dissected in PBS and fixed with 4% paraformaldehyde, 0.1% deoxycholate, and 0.1% Triton X-100 in PBS for 25 min at room temperature. They were blocked in PBS, 1% BSA, and 0.3% Triton for 1 h, incubated with the primary antibody over night at 4 °C, washed four times in washing buffer (PBS 0.3% Triton) and incubated with the appropriate fluorescent secondary antibodies for 1.5 h at room temperature in the dark. They were then washed and mounted in Vectashield (Cat# H-1000 RRID:AB_2336790) for confocal analysis.

TUNEL analysis was performed using In Situ “Cell Death Detection Kit” (TMR Red) (#12156792 910) and “Tunel Dilution Buffer” (#11966006001) kits, both from Roche.

All confocal images were obtained using a Leica LSM510 and LSM710 vertical confocal microscope. Multiple focal planes were obtained for each imaginal disc. Image treatment and analysis was performed using Fiji (https://fji.sc) and Adobe Photoshop software.

For the quantification of Dcp1, GFP, and lacZ staining, a Z-maximal intensity projection was generated for each image and a high-intensity threshold was adjusted for each image. Then, we calculated the percentage of staining covered in the region of interest.

The mitotic index was calculated as the average value of the ratio between the number of cells in mitosis (pH3-positive cells) and the area defined by the domains of expression the *sal*>*GFP*. The number of pH2AV foci was calculated similarly.

For the Fly-FUCCI cell quantification, third instar larvae of the a*p-Gal4;* UAS*-GFP-E2F11-230*, UAS*-mRFP1-NLS-CycB1-266*, genotype were subjected to IR and dissected at 1, 3, and 6 h after treatment. Nonirradiated larvae were used as control. Red, green, or yellow cells were manually quantified. For each experiment, at least five wing imaginal discs were used to count an average of 500 cells per disc. The same region of the imaginal disc for each disc was selected for the quantification.

The number of discs analyzed in each experiment is given in the figure legends.

Statistical analysis was performed using GraphPad Prism software (https://www.graphpad.com). The specific statistical test and the *n* used in each analysis are noted in the corresponding figure.

### Fluorescence-activated cell sorting

A total of 50 wing discs were dissected from third instar larvae expressing the corresponding transgene under the *sal*>*GFP* or *nub-Gal4* driver, depending on the experiment. Larvae were incubated for 40 min at 28 °C in 300 µl of trypsin solution (trypsin-EDTA, Sigma T4299) containing 1 µl of Hoechst (Hoechst 33342, Molecular Probes) in agitation. Trypsin digestion was stopped by the addition of 200 µl of 1% fetal bovine serum (FBS, Sigma 9665) in PBS. After centrifugation at 1500 × *g* at 4 °C for 5 min, cells were suspended in 300 µl of 1% FBS and cells were sorted by GFP expression using FACSVantage SE (BD Biosciences). The cell cycle profiles of GFP-positive and GFP-negative cells were determined by Hoechst fluorescence using a FACSCalibur flow cytometer (Becton Dickinson). The cell cycle profile was analyzed using FlowJo 7.5 software and Dean–Jett–Fox model.

### IR treatments

Third instar larvae of the indicated genotypes were irradiated in X-ray machine Phillips MG102 at the standard dose of 4000R and dissected at the indicated times depending on the experiment and stated in each figure.

### Comet assay for wing imaginal disc cells

DNA strand breaks and alkali-labile sites were assessed via the alkaline version of the Comet assay. A total of 60 wing imaginal discs cells were enzymatically individualized by incubation 20 min in TrypLE^TM^ Express Enzyme (Thermo Fisher Scientific, Waltham, Massachusetts, USA) and stored at −80 °C in freezing buffer (85.5 g/l sucrose and 50 mL/l DMSO prepared in 11.8 g/l citrate buffer at pH 7.6) until use.

An estimated 104 cells were embedded in 0.75% low melting point agarose and deposited on precoated slides with 1% agarose. Immediately after agarose solidification (10 min on ice), samples were incubated for 1 h at 4 °C in a cold lysis buffer (2.5 M NaCl, 100 mM EDTA, 10 mM Tris, 1% Triton X-100, pH10). The slides were then rinsed in 0.4 M Tris, pH 7.4. Subsequently, DNA was allowed to unwind for 40 min in the electrophoresis buffer (300 mM NaOH, 1 mM EDTA, pH > 13) and electrophoresis was carried out for 30 min at 25 V and 300 mA (0.73 V/cm). Slides were neutralized in 0.4 M Tris pH 7.4 and stained with 50 μl of GelRed (Thermo Fisher Scientific). Samples were examined with a Leica DMI 3000B microscope (Germany), equipped with an EL6000 compact light source and a 480–550 nm wide band excitation filter and a 590-nm cut-off filter. Scoring was carried out using the OpenComet plugin for the image-processing platform ImageJ. A total of 250 randomly selected cells were analyzed per condition. The tail moment (tail intensity × length summed over the whole extent of the tail) was used to measure DNA damage.

### BiFC assay

We used a pUASTattB that have the N-terminal (VN: 1-173) and C-terminal (VC: 155–238) moieties of Venus cloned in Xho1 and Xba1 restriction sites. The coding region of p53-A and Cdk1 were PCR amplified from the GH11591 clone (BDGP) and LD38718 clone (BDGP), respectively. Inserts were cloned in Xho1-Xba sites into the pUASTattB VN or VC version including a five amino acids linker region. N- and C-terminal deletions of p53-A were cloned in a similar manner. To ensure similar expression levels, all UAS constructs were inserted into the same attP site (86Fb), except UAS-VC-Cdk1 that was also inserted in 51D.

The sequence of all primers used in this study are as follows:

UAS-VN-p53-A:

Forward:

5´-CAGT**CTCGAG**GGCGGCTCAGGCGGCATGTATATATCACAGCCAATGTCGTGGC-3´.

Reverse: 5´-CAGT**TCTAGA**TCATGGCAGCTCGTAGGCACG-3´.

UAS-VN-p53-A^ΔN (1-83)^

Forward: 5´-CAGT**CTCGAG**GGCGGCTCAGGCGGCATGGAGAATCACAACATCGGTGG-3´.

Reverse:

5´-CAGT**TCTAGA**TCATGGCAGCTCGTAGGCACG-3´.

UAS-VN-p53-A^ΔC (283–385)^

Forward: 5´-CAGT**CTCGAG**GGCGGCTCAGGCGGCATGTATATATCACAGCCAATGTCGTGGC-3´.

Reverse:

5´-CAGT**TCTAGA**TCAGGACTTGCGCTTCTTGCTATTGAGCTGGCG-3´.

UAS-VC-Cdk1:

Forward: 5´-CAGT**CTCGAG**GGCGGCTCAGGCGGCATGGAGGATTTTGAGAAAATTG-3´.

Reverse: 5´-CAGT**TCTAGA**TTAATTTCGAACTAAGCCCGATTGAAAAC-3´.

For the initial BiFC analysis, we used the UAS-p53-VN flies available at FlyORF (F004757).

Visualization and quantification of the BiFC signal was done using identical parameters for image acquisition between the different genotypes and analyzed using Fiji.

### ChIP and quantitative real-time PCR assay

The following genotypes were used: *sal*>*GFP* (negative control)*; sal*>*GFP*, *p53-A (Myc)*, *miRHG* (positive control)*; sal*>*GFP, p53-A (Myc), Cdk1-i* (experimental condition); *sal*>*GFP, p53-A (Myc), dap* (experimental condition) and *sal*>*GFP, p53-A (Myc), fzr* (experimental condition). The wing imaginal discs of 100 larvae were dissected for each condition, performing three replicates per ChIP. Larvae were fixed in FA fix solution (1.8% formaldehyde, 50 mM HEPES pH 8, 1 mM EDTA, 0.5 mM EGTA pH 8, 100 mM NaCl) for 25 min at RT. Then, the tissue was incubated with Quench Buffer (1X PBS, 0.125 M glycine, 0.01% Triton X-100) for 6 min at RT. Larvae were washed with buffer A (10 mM HEPES pH8, 10 mM EDTA pH 8, 0.5 mM EGTA pH 8, 0.25% Triton X-100) and buffer B (10 mM HEPES pH 8, 200 mM NaCl, 1 mM EDTA pH 8, 0.5 mM EGTA pH 8, 0.01% Triton X-100) consecutively, 20 min each at 4 °C. Both A and B buffers were supplemented with 1 mM PMSF and 1X protease inhibitors cocktail (Roche #11873580001).

Wing imaginal discs were dissected in Buffer B on ice. Later, discs were centrifuged at max speed for 3 min at 4 °C. Collected disc pellet that was resuspended in buffer C (10 mM HEPES pH 8, 1 mM EDTA pH 8, 0.5 mM EGTA pH 8) supplemented with 1 mM PMSF and 1X protease inhibitors cocktail (Roche #11873580001). The discs were homogenized in this medium before proceeding to sonication. The tissue was sonicated 20 cycles (30″ ON/30″ OFF), at high power and at 4 °C using diagenode bioruptor sonicator. We removed 10% from the samples for input. Samples were precleared with protein G affinity gel (Sigma-Aldrich #E3403) for 1 h on rotator at 4 °C, and then the chromatin was transferred to a fresh tube. The anti-myc affinity gel (Sigma-Aldrich #E6654) and the protein G affinity gel (Sigma-Aldrich #E3403) as negative control of each chip were blocked in 1X RIPA (140 mM NaCl, 10 mM HEPES pH 8, 1 mM EDTA pH 8, 1% glycerol, 1% Triton X-100, 0.1% DOC) supplemented with 100 μg/ml salmon sperm DNA and 100 μg/ml BSA overnight at 4 °C.

Next day, we pelleted beads at 6000 rpm for 2 min and mixed the chromatin with the blocked beads. The samples were incubated for 4 h at 4 °C. Finally, the beads were washed four times in RIPA 1X for 5 min at 4 °C, pellet at 6000 rpm for 2 min at 4 °C. Beads were washed again in TE for 5 min at 4 °C and pellet at 6000 rpm for 2 min at 4 °C

Chromatin was eluted from beads in TE with 1% SDS and 0.1 M NaHCO_3_ at 50 °C.

To reverse the crosslinks, we incubated the eluted material at 65 °C overnight, processing the INPUT of each sample in parallel. The next day, we added 50 μg/ml of RNAase and incubated the samples 30 min at 37 °C. Then, 20 μg of proteinase K was added and incubated at 55 °C for 3 h. We added 4 μl NaCl 5 M and 4 μl Tris 1 M to each sample before purifying them by phenol/chloroform method. Finally, we added 1.25 μl of glycogen (Roche #10901393001), 25 μl of NaAC 3 M pH 5.2, and 550 μl of ethanol 100% at −20 °C ON. Samples were spin for 20 min at maximum speed and the pellet was twice with ethanol 70% at −20 °C and centrifuged again for 10 min and let the pellet dry at RT. The samples were resuspended in 30 μl of TE buffer and amplified by qPCR using GoTaq qPCR Master Mix (Promega #A6001), using as amplicons the p53^RE^ for the *rpr* and *hid* genes. Results were quantified using the delta Ct method and presented as percentage of input.

The sequence of the primers used in this study were previously described in ref. [39]:

*rpr*-p53-RE:

Forward: 5´-CTACGTTTCCCAGACCCAAGAC-3´.

Reverse: 5´-GTCTCCATCCAATTCCCATCTC-3´.

*hid*-p53-RE:

Forward: 5´-ACTTTTGTTCTTTTCGCTTTGGAC-3´.

Reverse: 5´-GATGACGAAATTCAAGCACACTCT-3´.

### ATAC-seq library preparation, sequencing, and data analysis

To carry out the ATAC-seq experiment, at least 30 wing discs were dissected from the following genotypes: *tub*^*Gal80*^>*GFP, tub*^*Gal80*^>*Cdk1-i*, and *tub*^*Gal80*^>*fzr*. Larvae were kept at 17 °C for 7 days and then transfer at 31 °C for 2 days before dissection. Two biological replicates for each condition were performed.

Samples were lysed in lysis buffer (10 mM Tris-HCl, pH 7.4, 10 mM NaCl, 3 mM MgCl2, 0.1% NP40). Lysates were centrifuged at 500 × *g* for 5 min at 4 °C to isolate the nuclei. Typically, we used 75,000 cells per condition. Next, samples were resuspended in 25 μl of transposition reaction mix as described in ref. [84] and incubated for 30 minutes at 37 °C.

Following the transposition, samples were purified using the Qiagen MinElute Kit following the manufacturer’s instructions.

ATAC-seq libraries were generated according to the standard protocol [84]. Briefly, transposed DNA fragments were amplified by a conventional PCR (5 min at 72 °C, 2.5 min at 95 °C, the thermocycling: X cycles of 20 s at 98 °C, 15 s at 63 °C, and 1 min at 72 °C) with Nextera barcoded primers. The number of cycles was empirically determined following Buenrostro et al.’s protocol [84]. Libraries were purified using a Qiagen MinElute Kit and eluted in 20 μl elution buffer. All the libraries were sequenced on an Illumina HiSeq 2500 (2 × 50 bp) according to the manufacturer’s instruction. Sequencing was performed at the Centre Nacional Anàlisi Genòmica (CNAG-CRG) sequencing facility in Barcelona, Spain.

ATAC-seq data analysis was performed using the nf-core/atacseq pipeline (v1.2.1), which runs Nextflow (v20.10.0), for quality controls, read alignment against Dm6 assembly using BWA-MEM (v0.7.17), filtering for blacklisted regions, data visualization, peak calling using MACS2 (v2.2.7.1), read count using featureCounts (v2.0.1), and differential accessibility analysis using the DESeq2 R library.

### Statistical analysis

Results were analyzed and presented using Prism 8 GraphPad software. We used unpaired two-tail Student’s *t* test when comparing the mean of two conditions or one-way ANOVA Dunnett’s test for multiple comparisons more. *P* values shown on the graphs are indicated with the following asterisk code: 0.05 (*), 0.01 (**), 0.001 (***), and 0.0001 (****). Sample size was described in each figure legend and in the corresponding “Materials and methods” section.

## Supplementary information

Supplementary Figure Legends

S1 fig

S2 Fig

S3 Fig

S4 fig

S5 Fig

S6 Fig

S7 Fig

S1 table

S2 Table

## Data Availability

ATAC-seq data from this study have been submitted to the NCBI Gene Expression Omnibus (http://www.ncbi.nlm.nih.gov/geo/) under accession number GSE169668.
