# Supplementary material for: Coordination between cell proliferation and apoptosis after DNA damage in Drosophila
Source: Cell Death Differ. 2021 Nov 25;29(4):832–45. doi: 10.1038/s41418-021-00898-6 (PMC8989919; doi:10.1038/s41418-021-00898-6)
Supplement: Supplementary file 1 — Supplementary Figure Legends [file 41418_2021_898_MOESM1_ESM.docx]

**Supplementary figure and table legends**

**S1 Fig: Analysis of cell cycle arrest tools.**

A) Cell cycle perturbations by the expression of *CycE-i*, *E2f1-i*, *Rbf^280^* and *CycA-i* with the *sal-Gal4* (*sal>*) driver. The Fly-FUCCI system (*ubi-GFP-E2F11-230* and *ubi-mRFP1-NLS-CycB1-266*) and pH3 staining (blue) was used to visualize the cell cycle. Scale bar: 50 μm.

B) Adult wing phenotypes of the experiments presented in A are shown. The *sal* domain is colored in green in the control.

C) Cell cycle profiles of dissociated wing imaginal discs expressing the indicated cell-cycle regulators and GFP in the *sal* domain are shown. Red profiles correspond to control GFP negative cells and green profiles belong to GFP positive cells in control and cell cycle perturbed cells.

D) Mitotic index measured as the number of pH3 positive cells per area in the *sal* domain of control (*sal>GFP*) and in cell cycle arrested cells and endocycle-induced cells. n>11 discs per genotype. Error bars indicate standard error of the mean (SEM). **** P value <0,0001 by one-way ANOVA when compared the mean of each column with the mean of the control.

**S2 Fig: IR-induced apoptosis analysis in cell cycle arrested cells.**

A) GFP (green), Dcp1 (red and white) and Topro-3 (blue) staining wing imaginal discs expressing the indicated transgene by the *sal-Gal4*. Below each panel, the Dcp1 channel is shown and the *sal* domain is outlined by green dotted lines.

B) GFP (green) and TUNEL staining (red and white) in wing imaginal discs expressing the indicated transgene by the *sal-Gal4* in irradiated discs and analyzed 24 hrs later. Below each panel, the TUNEL channel is shown and the *sal* domain is outlined by green dotted lines.

C) Non-irradiated and irradiated wing imaginal discs expressing the indicated transgenes under the *sal-Gal4* driver. Discs were dissected 24 hrs after IR and stained for Dcp1 (red and white) and GFP (green). Below each panel, the Dcp1 channel is shown and the *sal* domain is outlined by green dotted lines.

D) Wing imaginal discs expressing the indicated transgene by the *sal-Gal4* in control discs and irradiated discs analyzed 4 hrs later. GFP is in green and Dcp1 staining in red and white. The Dcp1 channel is shown and the *sal* domain is outlined by green dotted lines.

Scale bar: 50 μm.

**S3 Fig: Apoptotic response after IR in cell cycle arrested and endocycle-induced cells that simultaneously knockdown DNA repair mechanisms.**

A) Strong pH2Av foci (green and arrows) are associated to cells with high levels of Dcp1 (red and white) in non-irradiated and irradiated wing imaginal discs. Separate channels for Dcp1 staining are shown.

B) Irradiated wing imaginal discs analyzed 4 hrs later expressing the *mre11-i* line and the indicated transgenes by the *sal-Gal4, UAS-GFP* driver. GFP is green and Dcp1 staining is red or white. Below each panel, the Dcp1 channel is shown and the *sal* domain is outlined by green dotted lines. B’) Quantification of Dcp1 staining in the *sal* domain of wing imaginal discs of the corresponding genotypes presented. Error bars indicate the minimum and maximum point for each genotype. Individual wing discs measurements are shown. n>15 discs per genotype. **** P value <0,0001 by one-way ANOVA when compared the mean of each column with the mean of the corresponding control. ns, not significant.

C) Irradiated wing imaginal discs analyzed 4 hrs later expressing the indicated transgenes by the *sal-Gal4, UAS-GFP* driver in a *mei-41^D5^* mutant background. GFP is green and Dcp1 staining is red or white. Below each panel, the Dcp1 channel is shown and the *sal* domain is outlined by green dotted lines. C’) Quantification of Dcp1 staining in the *sal* domain of wing imaginal discs of the corresponding presented genotypes. Error bars indicate the minimum and maximum point for each genotype. Individual wing discs measurements are shown. n>15 discs per genotype. **** P value <0,0001 by one-way ANOVA when compared the mean of each column with the mean of the corresponding control. ns, not significant.

D) Irradiated wing imaginal discs analyzed 4 hrs later expressing the *tefu-i* line and the indicated transgenes by the *sal-Gal4, UAS-GFP* driver. GFP is green and Dcp1 staining is red or white. Below each panel, the Dcp1 channel is shown and the *sal* domain is outlined by green dotted lines. D’) Quantification of Dcp1 staining in the *sal* domain of wing imaginal discs of the corresponding presented genotypes. Error bars indicate the minimum and maximum point for each genotype. Individual wing discs measurements are shown. n>15 discs per genotype. **** P value <0,0001 by one-way ANOVA when compared the mean of each column with the mean of the corresponding control. ns, not significant.

Note that cell cycle arrested and endocycle induced-cells attenuate apoptosis in *mei-41^D5^* mutants and Mre-11 and Tefu depleted cells to the same extent as their corresponding controls.

Scale bar: 50 μm.

**S4 Fig: Analysis of Hid and Rpr activity in cell cycle arrested and endocycle-induced cells.**

A) Wing imaginal discs expressing the indicated transgenes under the *sal-gal4, UAS-GFP* driver and the *hid^20-10^* regulatory region driving the *lacZ* reporter gene (*hid^20-10^-Z*) (red and white). Note that the *hid^20-10^-Z*  is weakly active in the wing pouch in non-irradiated discs, however it is strongly activated 24 hrs after IR. *hid^20-10^-Z* separate channel is shown below each panel and the *sal* domain marked by green dotted lines.

B) Dcp1 staining (red) in wing imaginal discs expressing *rpr* and the indicated transgenes under the *sal-Gal4, UAS-GFP* driver. GFP is in green, Dcp1 in red and Topro-3 in blue.

Scale bar: 50 μm.

**S5 Fig: Analysis of p53 protein levels in cell cycle arrested and endocycle-induced cells of irradiated discs.**

Irradiated third instar wing imaginal discs expressing the indicated transgene with by the *hh-Gal4, tub-Gal80^ts^* (*hh^Gal80^>*) driver and stained for p53 (red and white) and GFP (green). Separate channel for p53 is shown below each image. The antero-posterior compartment boundary is marked by a green dotted line. The gain in the p53 channel has been increased for visualization purposes. Larvae were kept at 17ºC and shifted to 31ºC for 24 hrs and wing discs were dissected 4 hrs after IR. Although a slight decrease of p53 protein levels could be observed in posterior cells expressing *dap*, no changes were detected for the other cell cycle modifications.

Scale bar: 50 μm.

**S6 Fig: IRER activity in cell cycle arrested and endocycle-induced cells.**

A) Analysis of p53-A binding by chromatin immunoprecipitation experiments with anti-Myc at the p53^RE^ of the *hid* and *rpr* genes from wing imaginal discs of the following genotypes:

*-sal>GFP*

-*sal>GFP*, *p53-A (Myc)*, *miRHG*

-*sal>GFP, p53-A (Myc), dap*

-*sal>GFP, p53-A (Myc), Cdk1-i*

-*sal>GFP, p53-A (Myc), fzr*

Enrichment values were normalized to a ‘mock’ sample (IgG). Error bars represent SEM of three independent experimental replicates.

B) Wing imaginal discs expressing the indicated transgenes under the *sal>* driver and *hid 5´F* regulatory region driving the *GFP* reporter gene (*hid 5´F-GFP*). *hid 5´F-GFP* activity is in green, p53 in red and Topro-3 in blue. Separate channel for the *hid 5´F-GFP* is presented below each image with the *sal* domain marked with a red dotted line. All the images were taken keeping the same confocal settings.

C) Quantification of *hid 5´F-GFP* staining in the *sal* domain of wing imaginal discs from the genotypes presented in B. Error bars indicate SEM. n>10 disc per genotype. **** P value <0,0001 by one-way ANOVA when compared the mean of each column with the mean of the control (*sal>p53-A*).

D) Wing imaginal discs expressing the indicated transgenes under the *sal>GFP* driver and *hid^20-10^* regulatory region driving the *lacZ* reporter gene (*hid^20-10^-Z*). *hid^20-10^-Z* activity is in red, GFP in green and Topro-3 in blue. Separate channel for the *hid^20-10^-Z* is presented below each image with the *sal* domain marked with a red dotted line. All the images were taken keeping the same confocal settings.

E) Quantification of *hid^20-10^-Z* staining in the *sal* domain of wing imaginal discs from the genotypes presented in D. Error bars indicate SEM. n>10 disc per genotype. **** P value <0,0001 by one-way ANOVA when compared the mean of each column with the mean of the control (*sal>GFP, p53-A*).

F) Schematic representation of the *Drosophila* H99 locus and the location of the irradiation responding enhancer region (IRER). The IRER contains a p53^RE^ and it is critical for apoptotic induction after irradiation. The accessibility of the IRER is subject to epigenetic regulation. An ubiquitin-DsRed reporter was inserted into IRER through homologous recombination (67).

G) Third instar wing imaginal discs carrying the IRER{ubi-DsRed} cassette (red and white) and expressing the corresponding transgenes under the *sal>GFP* driver (green). Separate channel for the IRER is presented below each image with the *sal* domain marked with a green dotted line.

Scale bar: 50 μm.

**S7 Fig: BiFC analysis of p53 interaction with Cdk1.**

A) Third instar wing imaginal discs expressing the indicated VC and VN fusion proteins under the *sal-Gal4* driver. Note that the strong BiFC signal is specific to the VC-Cdk1/p53-VN pair as the expression of two unrelated proteins failed to complement p53-A or Cdk1 as strong as the p53-A/Cdk1 complex. In addition, the expression of a “cold” competitive partner of p53-A, Cdk1-HA, led to a decrease to background levels of the VC-Cdk1/p53-VN BiFC signal.

B) Quantification of fluorescent signal resulting from the BiFC experiments. Data are represented as a boxplot showing each individual measurement. n>20 discs per genotype. Error bars indicate the minimum and maximum point for each genotype. **** P value <0,0001 by one-way ANOVA when compared the mean of each column with the mean of the corresponding control. All the images were taken keeping the same confocal settings.

Scale bar: 50 μm.

**S1 Table: ATAC-seq comparative analysis**

Different chromatin accessibility regions identified between wing imaginal discs of the following genotypes using the DESeq2 R library:

*tub^Gal80^>Cdk1-i* and *tub^Gal80^>GFP*

*tub^Gal80^>fzr* and *tub^Gal80^>GFP*

Each region was named with an ID (interval) and genomic coordinates are shown.

**S2 Table: Reagents and tools table**
