# Supplementary figures and images for: Coordination between cell proliferation and apoptosis after DNA damage in Drosophila
Source: Cell Death Differ. 2021 Nov 25;29(4):832–45. doi: 10.1038/s41418-021-00898-6 (PMC8989919; doi:10.1038/s41418-021-00898-6)

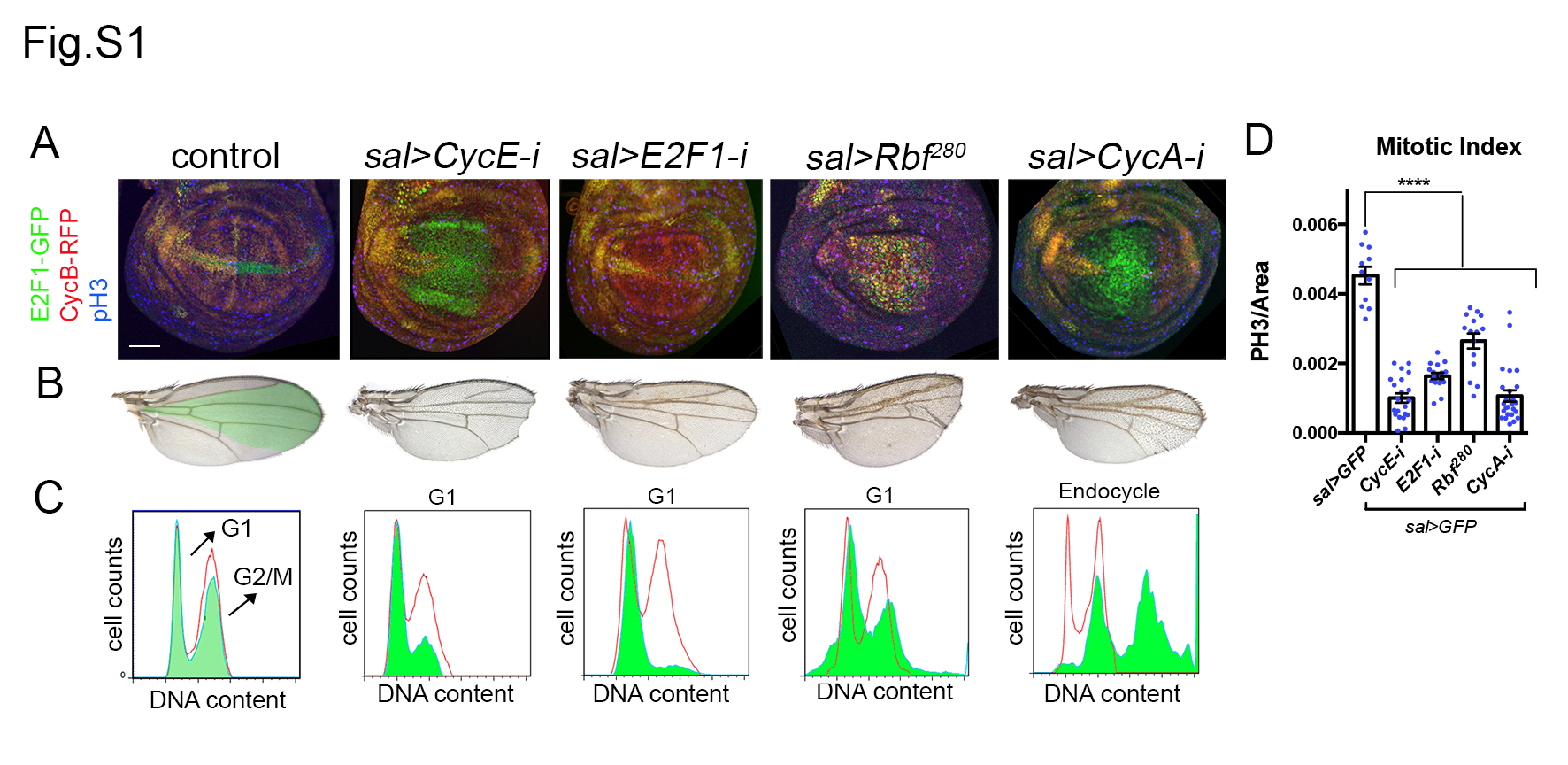

Supplement: Supplementary file 2 — S1 fig [file 41418_2021_898_MOESM2_ESM.tif]

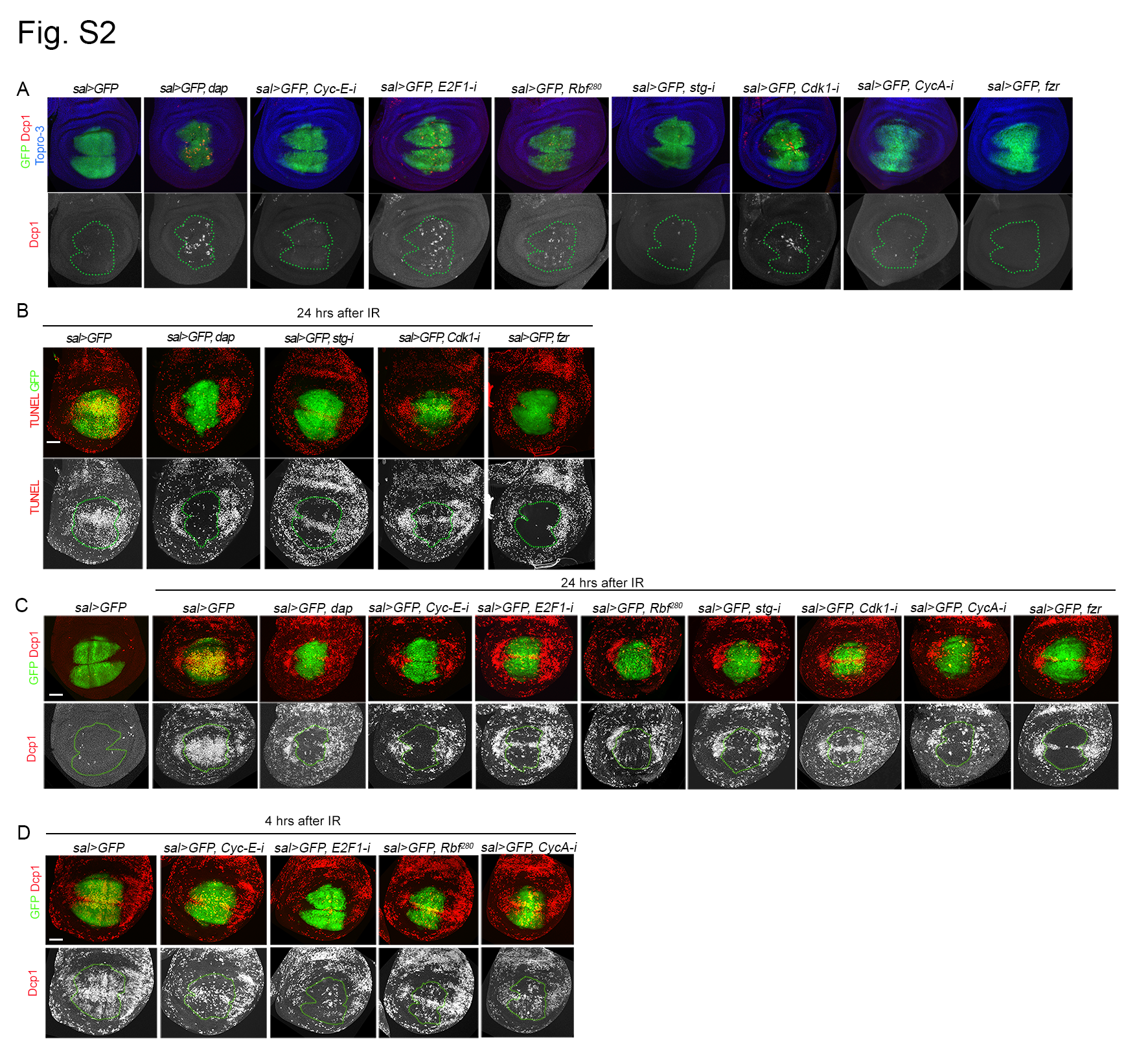

Supplement: Supplementary file 3 — S2 Fig [file 41418_2021_898_MOESM3_ESM.tif]

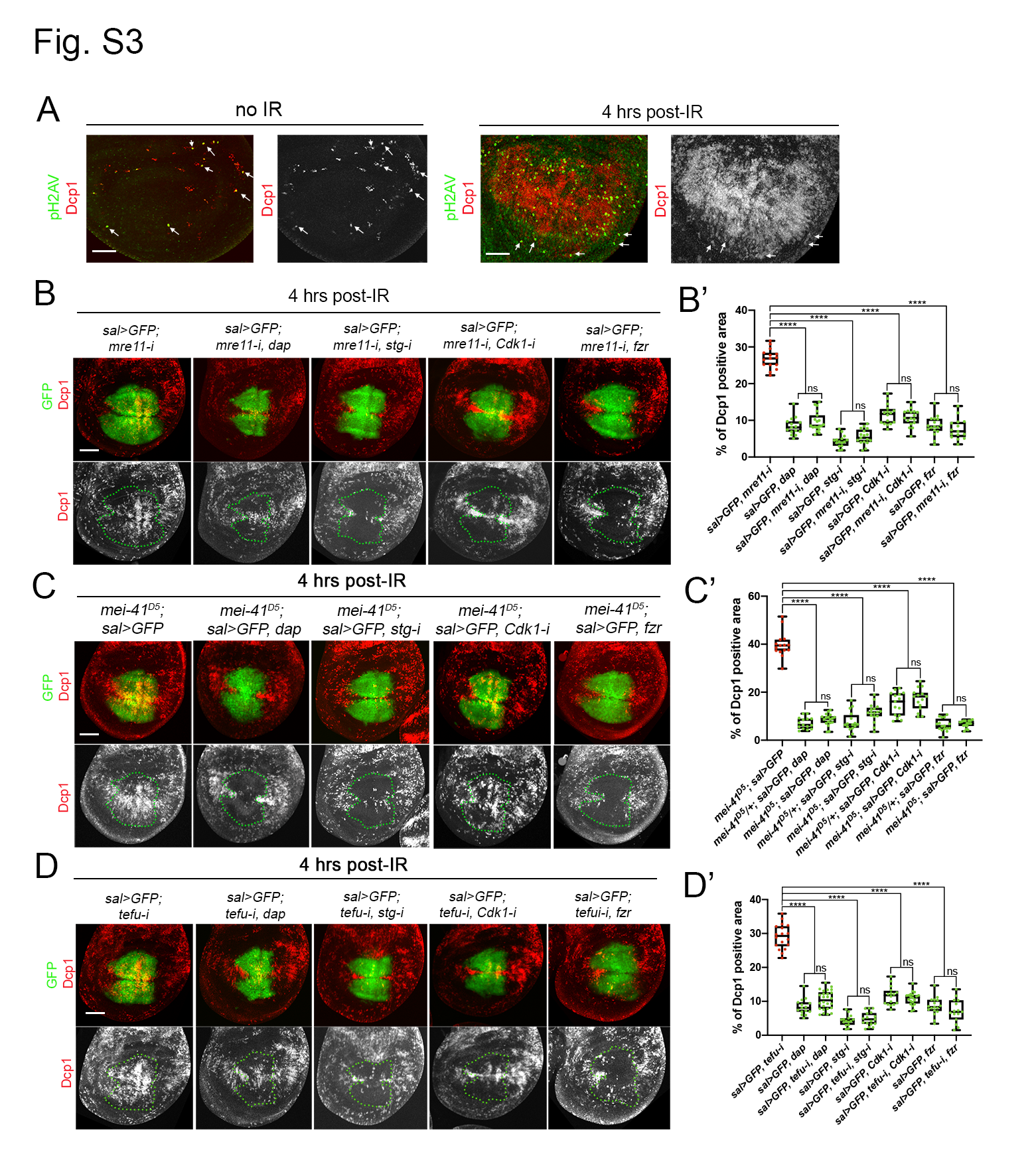

Supplement: Supplementary file 4 — S3 Fig [file 41418_2021_898_MOESM4_ESM.tif]

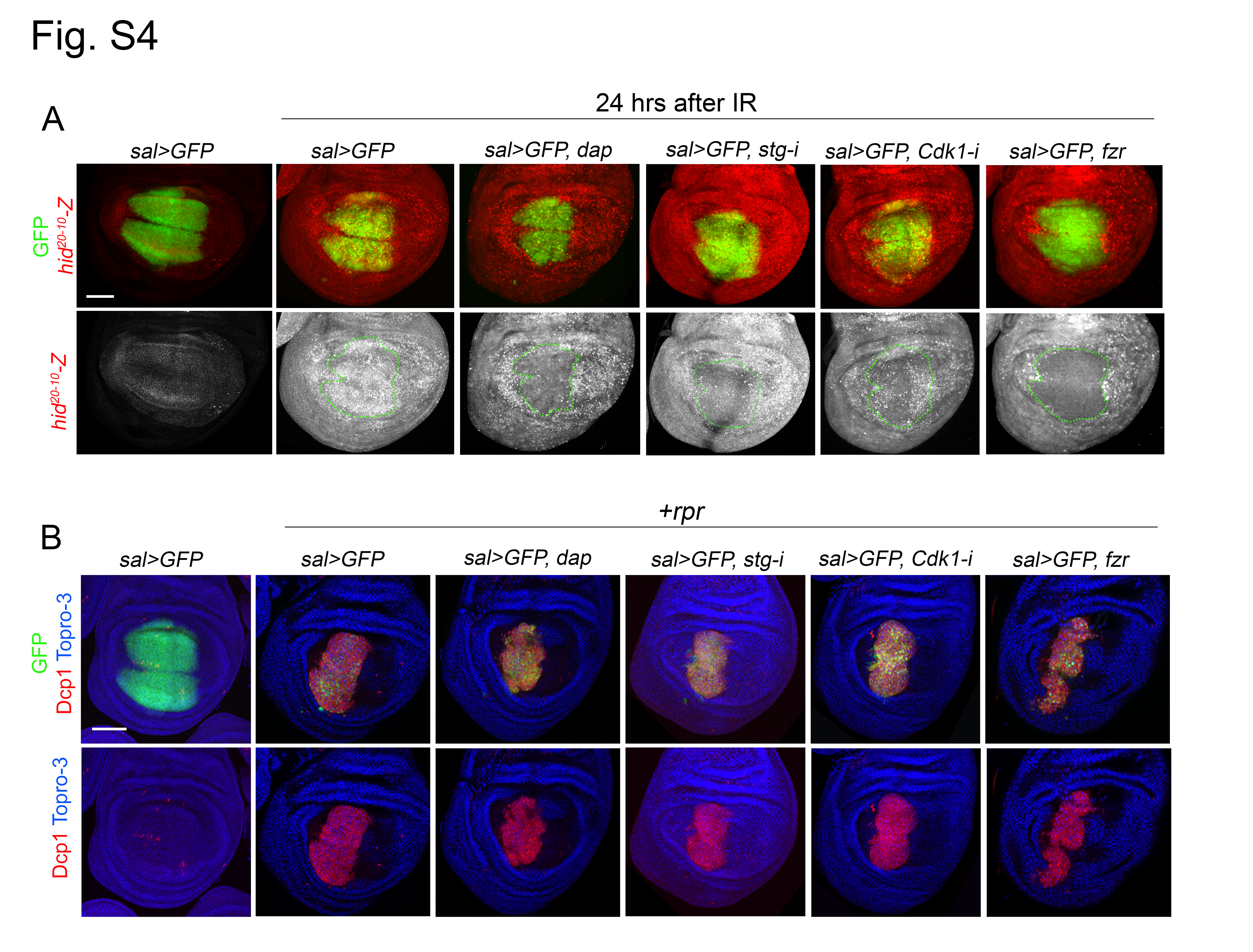

Supplement: Supplementary file 5 — S4 fig [file 41418_2021_898_MOESM5_ESM.tif]

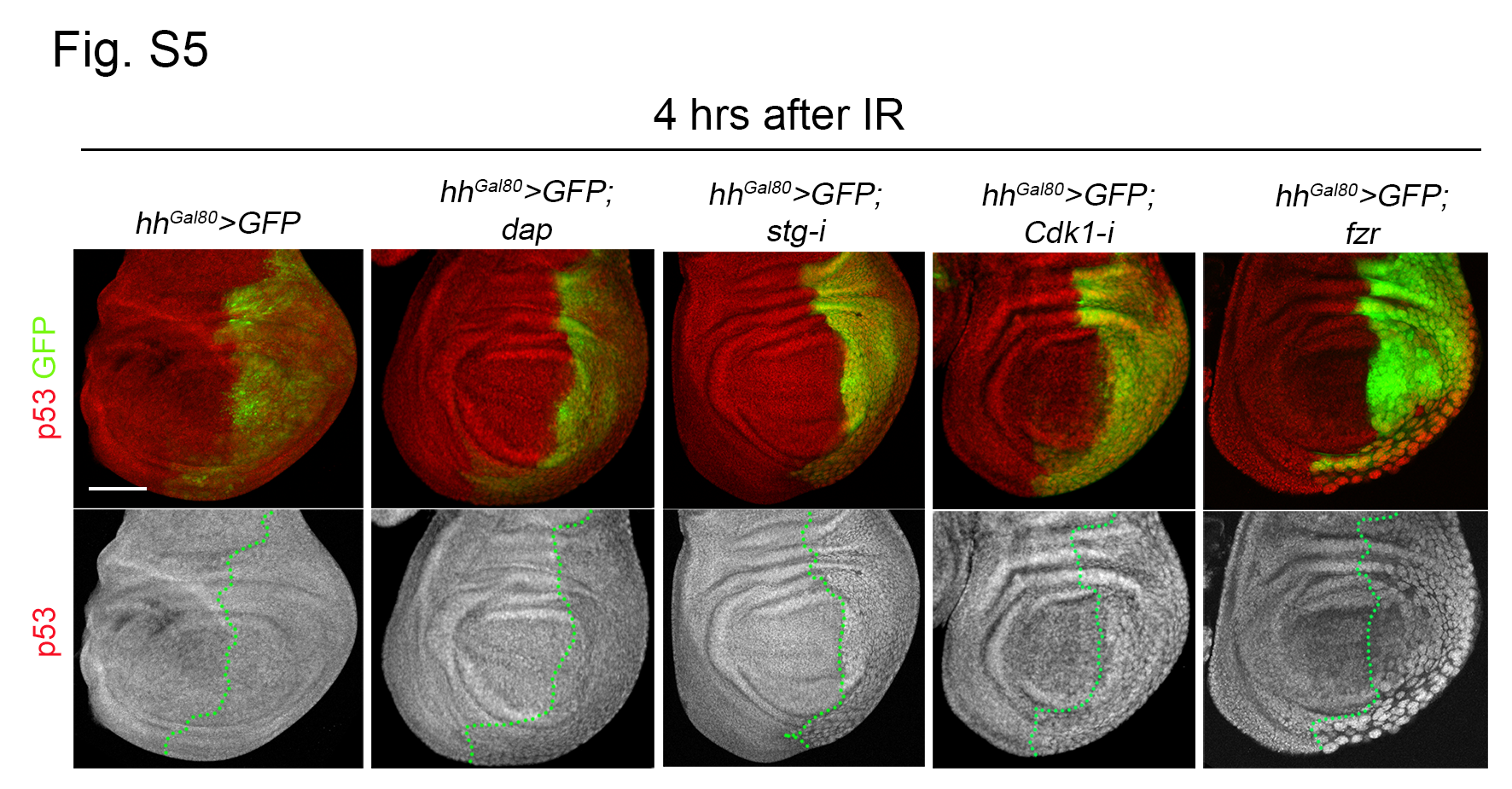

Supplement: Supplementary file 6 — S5 Fig [file 41418_2021_898_MOESM6_ESM.tif]

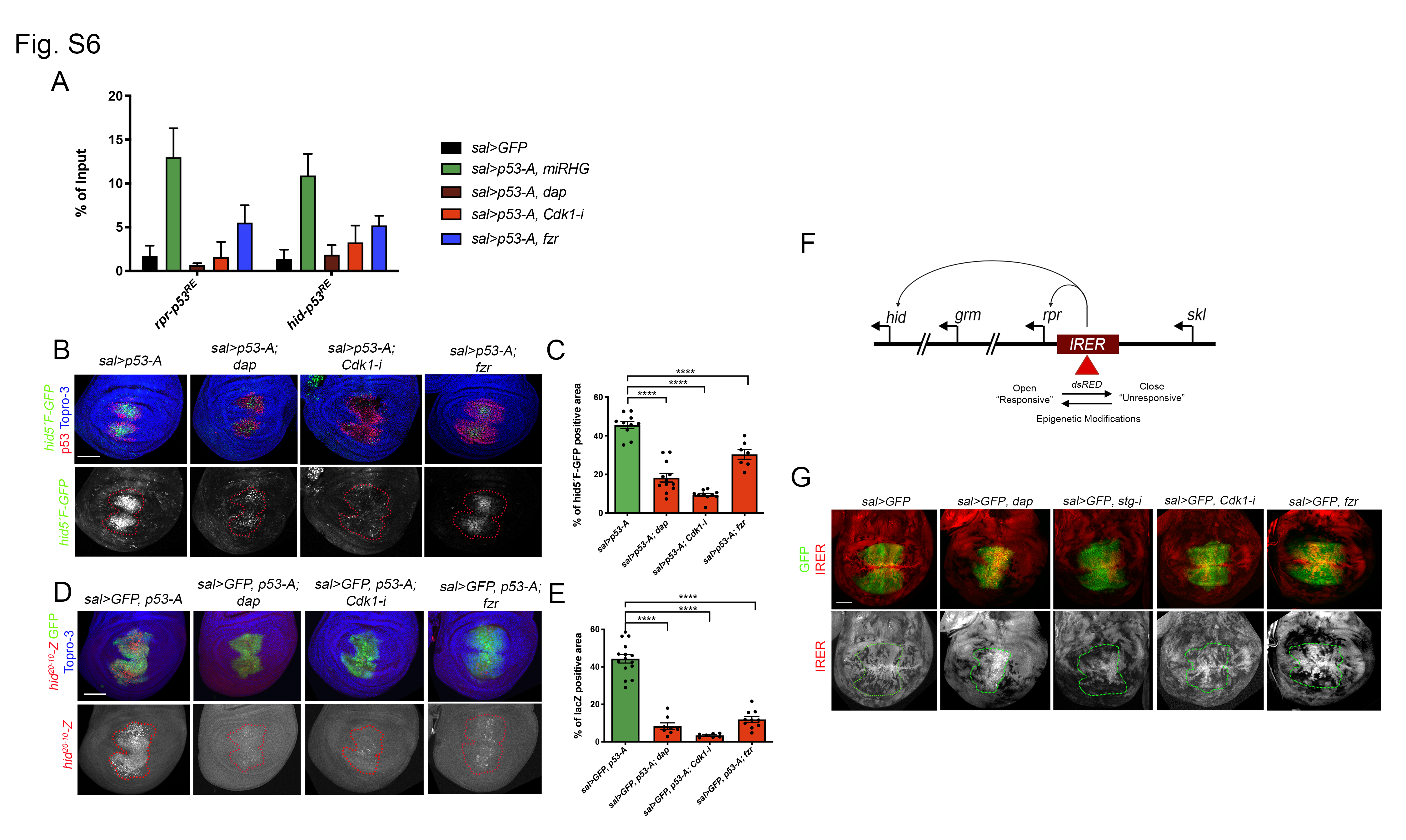

Supplement: Supplementary file 7 — S6 Fig [file 41418_2021_898_MOESM7_ESM.tif]

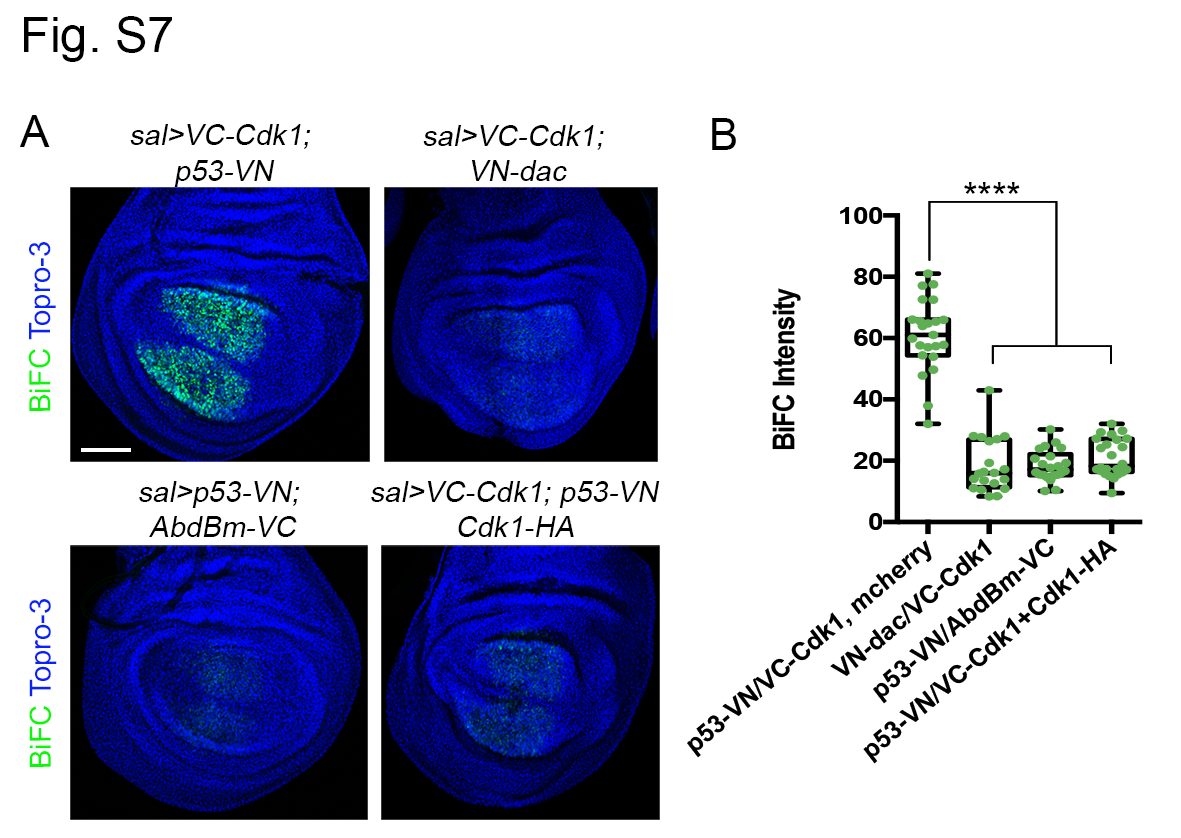

Supplement: Supplementary file 8 — S7 Fig [file 41418_2021_898_MOESM8_ESM.tif]
